# A Forgotten Corner in Cancer Immunotherapy: The Role of Lipids

**DOI:** 10.3389/fonc.2021.751086

**Published:** 2021-10-14

**Authors:** Yang Yu, Lei Gao, Yunpeng Wang, Bo Xu, Ewetse Paul Maswikiti, Haiyuan Li, Peng Zheng, Pengxian Tao, Lin Xiang, Baohong Gu, Alexandra Lucas, Hao Chen

**Affiliations:** ^1^ Department of Surgical Oncology, Lanzhou University Second Hospital, Lanzhou, China; ^2^ The Second Clinical Medical College, Lanzhou University, Lanzhou, China; ^3^ Key Laboratory of Digestive System Tumors of Gansu Province, Lanzhou University Second Hospital, Lanzhou, China; ^4^ Center for Personalized Diagnostics and Center for Immunotherapy, Vaccines and Virotherapy, The Biodesign Institute, Arizona State University, Tempe, AZ, United States

**Keywords:** lipids, fatty acids, tumour microenvironment, immunotherapy, immune evasion

## Abstract

In the past decade, cancer immunotherapy has achieved great success owing to the unravelling of unknown molecular forces in cancer immunity. However, it is critical that we address the limitations of current immunotherapy, including immune-related adverse events and drug resistance, and further enhance current immunotherapy. Lipids are reported to play important roles in modulating immune responses in cancer. Cancer cells use lipids to support their aggressive behaviour and allow immune evasion. Metabolic reprogramming of cancer cells destroys the equilibrium between lipid anabolism and catabolism, resulting in lipid accumulation within the tumour microenvironment (TME). Consequently, ubiquitous lipids, mainly fatty acids, within the TME can impact the function and phenotype of infiltrating immune cells. Determining the complex roles of lipids and their interactions with the TME will provide new insight for improving anti-tumour immune responses by targeting lipids. Herein, we present a review of recent literature that has demonstrated how lipid metabolism reprogramming occurs in cancer cells and influences cancer immunity. We also summarise the potential for lipid-based clinical translation to modify immune treatment.

## 1. Introduction

Since current immunotherapy has shown dramatic effects in controlling cancer, research into immune responses in cancer has attracted great interest. The limitations of current immunotherapy include a small beneficial population and unavoidable disease relapse. The rationale or foundation of immunotherapy is to design a strategy that promotes immune responses to tumour antigens.

**Figure 1:**
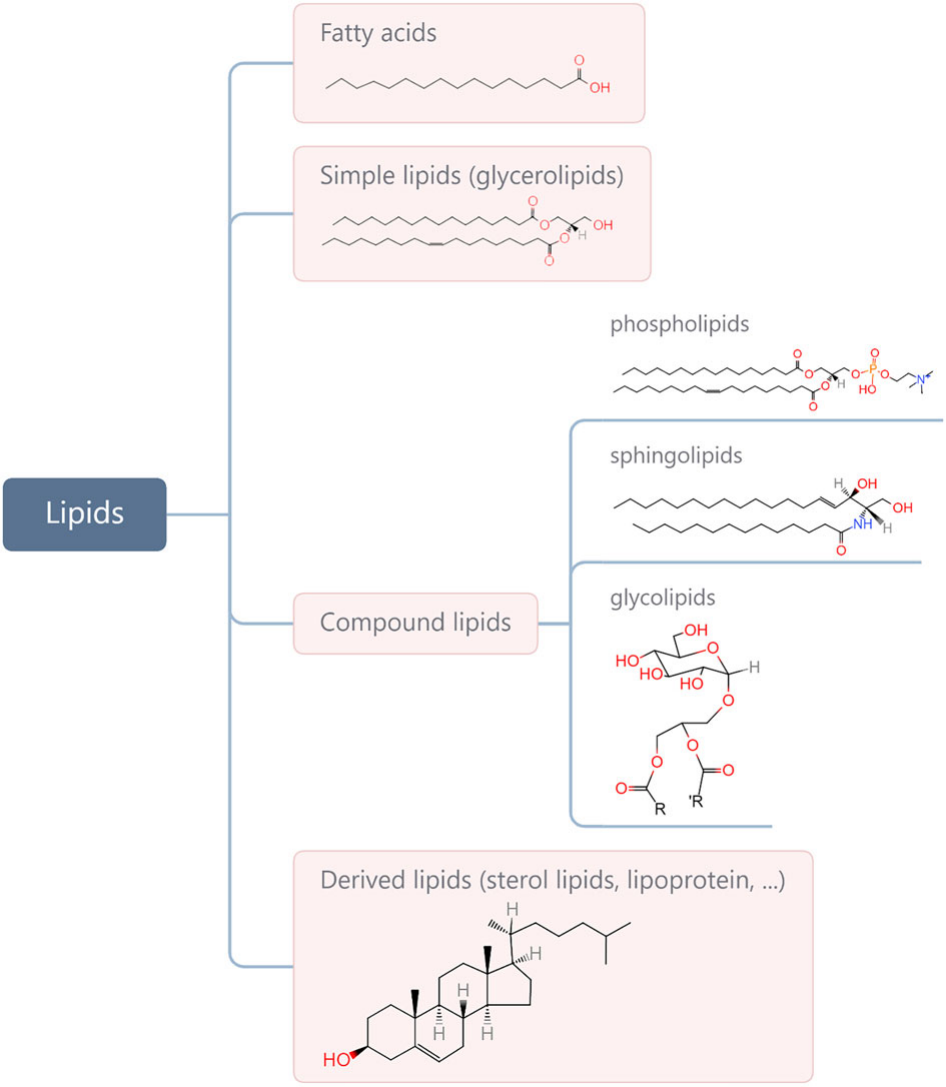
Categories of main lipids involved in cellular processes and representative structures from each category.

Lipids are a complex group of biomolecules with various compositions and functions, including fatty acids (FAs), glycerolipids, phospholipids, sphingolipids, glycolipids, sterol lipids, and lipoprotein. A simple method for biological lipid classification, with the representative structures, is shown in **Figure 1**. Many lipids are derived from FAs that are composed of long hydrocarbon chains with different lengths and saturation. Although most FAs (nonessential FAs) can be synthesised in the body, some must be obtained from diet (i.e., so-called essential FAs and including omega-3 and omega-6). Lipids play a central role in many normal cellular processes, and maintaining the physiological levels of lipids contributes to homeostasis throughout the body. Specifically, glycerophospholipids are the main structural components of the biological membranes that physically separate the intracellular components from the extracellular environment. Lipids are also a major form of energy storage, which is stored in the form of triglycerides in adipose tissue, and lipases catalyse the hydrolysis of triglycerides to produce FAs to fuel cell activity through β oxidation. In addition, lipids in the body are important signalling molecules and cellular messengers, such as sphingosine-1-phosphate, eicosanoids, and prostaglandins, *via* activation of G protein-coupled or nuclear receptors (1–3). Under tumour conditions, however, cancer cells hijack lipids to aid in their development and progress by promoting proliferation and invasion of cancer cells (4–9). Changes in lipid metabolism and related signalling have been recognised as hallmarks of cancer. In addition, lipids present within the tumour microenvironment (TME) play an important role in eliciting immune responses against cancer. A negative relationship between the function of lipids and anti-tumour responses has been widely reported (10–13). Therefore, understanding how cancer cells contribute to changes in lipid metabolism in the TME and are influenced in return, as well as how lipids take part in the interplay with cancer immunity, is of great significance for developing more effective interventions.

In this review, we discuss recent advances in the role of lipids in remodelling immune responses in cancer. At the same time, we also explore the potential clinical application of lipids as a new therapeutic approach or as biomarkers for cancer based on their immunoregulatory role. The search strategy for literatures of interest can be seen in the **Supplementary Material**.

## 2. Interaction Between Lipids and Tumour Cells

Lipids can act as substrates for the synthesis of cell membranes and organelles for cancer cell proliferation and are an important source for cancer cells to obtain ATP. In addition, lipids play an important role beyond the metabolic requirements, such as the role of mediator in signal transduction. In general, lipids in cancer cells are altered in density, composition, distribution, and mode of action. This significantly contributes to the development and progression of cancer. Next, we discuss how the metabolism and utilisation of lipids change in cancer cells and how these changes can aid in their malignant phenotypes.

### 2.1. Lipid Metabolism Reprogramming in Tumour Cells

**Figure 2:**
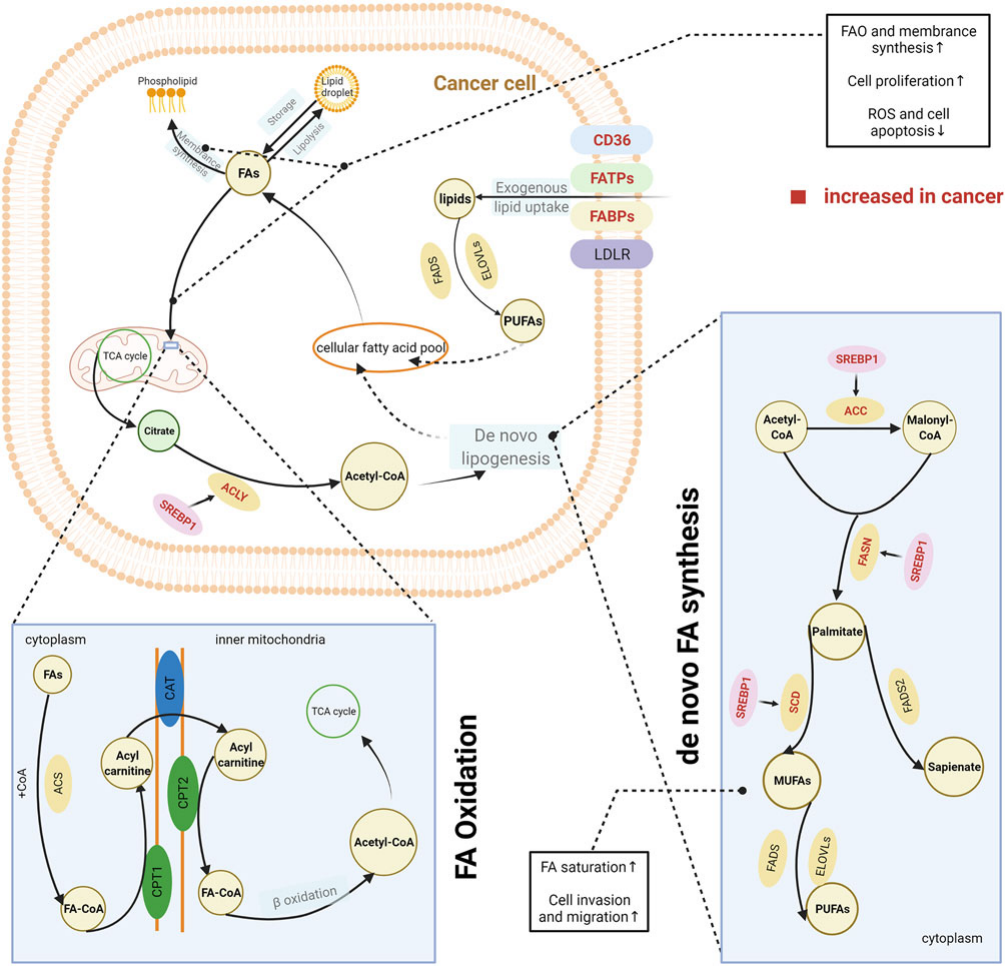
Lipid metabolism reprogramming in cancer cells. To satisfy the requirements for cancer cell survival and proliferation, cancer cells have an increased uptake of exogenous lipids and a high level of *de novo* synthesis. Exogenous uptake of lipids is increased in cancer cells through upregulating multiple transporters, including CD36, FATPs, and FABPs. *De novo* FA synthesis is activated in cancer cells. This is achieved through the overexpression of lipogenic enzymes, induced by the activation of SREBPs. Abundant lipids can support the malignant proliferation of cancer cells by providing necessary substrates for membrane synthesis and metabolic fuels *via* β-oxidation. Increased FAO can also help reduce cell apoptosis by eliminating reactive oxygen species. In addition, balance between saturated and unsaturated FAS was achieved by lipid reprogramming to enhance the capacity of invasion and migration in cancer cells. The excess lipids are stored into LDs. The figure was created with BioRender.com. ACC, acetyl-CoA carboxylase; ACLY, ATP citrate lyase; ACS, acyl-CoA synthetase; CAT, carnitine translocase; CD36, cluster of differentiation 36; CPT, carnitine palmitoyltransferase; ELOVL, fatty acid elongase; FA, fatty acid; FABPs, fatty acid-binding proteins; FA-CoA, fatty acyl-CoA; FADS, fatty acid desaturase (Δ5 or Δ6); FAO, fatty acid oxidation; FASN, fatty acid synthase; FATPs, transport proteins; LD, lipid droplet; LDLR, low-density lipoprotein receptor; MUFA, monounsaturated fatty acid; PUFA, polyunsaturated fatty acid; SCD, stearoyl-CoA desaturase (Δ9); SREPs, sterol regulatory element binding proteins; TCA cycle, tricarboxylic acid cycle.

Highly proliferative cancer cells demonstrate or have a significant dependence on lipids, such as phospholipids in synthesis of cell membranes and FAs in generation of ATP. Thus, cancer cells, unlike normal tissue cells, have special lipid metabolic features, known as lipid metabolism reprogramming. Cancer cells modify lipid metabolism to meet their survival needs by increasing the exogenous uptake of lipids or *de novo* lipogenesis (**Figure 2**).

CD36, a membrane-bound glycoprotein, plays an important role in delivering exogenous lipids into the cytoplasm of cells (14). CD36 was overexpressed on the cell membrane of several cancer cells and was associated with the aggressive behaviours of these cancers, including oesophageal and gastric cancer, breast cancer, cervical cancer, and renal cancer (15–19). In addition, other proteins such as FA transport proteins (FATPs) and FA-binding proteins (FABPs) also contribute to the uptake of exogenous lipids by cancer cells (20, 21). One source of exogenous lipids is FAs, which are released by adipocytes. Many studies have reported an interaction between cancer cells and the surrounding adipocytes. The coculture of breast cancer cells and adipocytes can activate lipolysis within adipocytes, and the released FAs are taken up and utilised by breast cancer cells (22). Both activated adipocytes and cancer cells secrete IL-6, which is considered a strong lipolytic factor that induces the release of FAs from adipocyte triglyceride stores (23–26). IL-6 delivers signals through the STAT3 pathway, and recent studies have reported that CD36 is a downstream target of activated STAT3, suggesting that upregulation of IL-6 expression would further increase the uptake of FAs by cancer cells (27, 28). Dietary sources are another way for cancer cells to acquire exogenous FAs. Triglycerides present in circulating very low-density lipoproteins (VLDL) can be hydrolysed by lipoprotein lipase (LPL), which is highly expressed in several types of cancer (29–32). In addition, receptor-mediated endocytosis of intact VLDL has recently been reported as a newly identified means for lipid uptake by cancer cells (33). In general, FAs are taken up by cancer cells *via* CD36, FATPs, or FABPs, whereas complex lipids are taken up *via* the low-density lipoprotein receptor (LDLR).

Apart from the exogenous uptake of lipids, *de novo* lipogenesis is a common feature of cancer cells (34). Somatic cells normally obtain FAs from exogenous sources, including diet or liver-synthesised lipids. In contrast, cancer cells also activate *de novo* lipogenesis pathways, which remove their reliance on externally derived lipids (34). FAs are synthesised from acetyl-CoA. Several key lipogenic enzymes, such as acetyl-CoA carboxylase (ACC), FA synthase (FASN), and stearoyl-CoA desaturase (Δ9) (SCD), participate in this process, and in cancer cells they are upregulated to increase *de novo* FA synthesis (35, 36). Increased expression of these enzymes was induced by the activation of sterol regulatory element-binding proteins (SREBPs), which are key transcription factors involved in lipid metabolism (37). SREBPs were mostly regulated by the PI3K/Akt/mTORC1 signalling axis (37). This axis increases the expression of enzyme need for FA synthesis therewith and activates the ATP-citrate lyase (ACLY) that catalyses the generation of acetyl-CoA from citrate. Therefore, it was earlier widely accepted that *de novo* lipogenesis was a universal phenotypic alteration for cancer cells irrespective of the surrounding levels of circulating lipids. The synthesised FAs are ultimately used for production of phospholipids for membranes and lipid rafts by cancer cells. However, some studies have challenged this perspective, as they have found that these newly synthesised FAs are beyond the requirements of membrane synthesis in cancer cells (38, 39). *De novo* lipogenesis changes the cellular lipid composition in cancer cells compared with normal tissues (40), which can be used as a marker to detect cancer; it also adjusts the saturation of membrane lipids, which is an important factor in influencing cell dynamics and the susceptibility to peroxidation. *In vitro* and *in vivo* evidence has shown that increase of *de novo* lipogenesis can increase the level of saturated and mono-unsaturated phospholipid species but decrease the relative amount of polyunsaturated FAs (PUFAs), which are obtained through exogenous uptake (41). Because saturated lipids pack more densely and PUFAs are more susceptible to peroxidation, this change of increased saturation assists the maintenance of cancer cell shape and motility and protects cancer cells from lipid peroxidation in the presence of reactive oxygen species. Reversely, the inhibition of *de novo* lipogenesis *via* lipogenesis inhibitors or by targeting lipogenic enzymes with small interfering RNA can dramatically alter the membrane dynamics and render cancer cells more susceptible to oxidative stress-induced cell death (41).

Taken together, cancer cells have an increased level of intracellular lipids through either or both excessive exogenous uptake and *de novo* synthesis. The choice of the FA source for cell biosynthesis likely depends on the conditions within the TME. For example, in a hypoxic TME, cancer cells may bypass lipid synthesis pathways and increase the uptake of exogenous unsaturated FAs (42). Cancer cells can utilise these synthesised or extraneous FAs through multiple mechanisms to achieve their growth and gain a survival advantage. Naturally, an increased amount of FAs can aid in membrane synthesis and energy supply in the life cycle of cancer cells (4–6), promoting the proliferation of cancer cells as a result. Beyond the biosynthesis requirements, increased FAs can support a more aggressive phenotype in cancer cells (43). It was reported that FA oxidation (FAO) contributed to reduction of reactive oxygen species and cell apoptosis in acute monocytic leukaemia cells (44). In addition, as mentioned above, the balance between saturated and unsaturated FAs is critical in maintaining the cell shape and capacity of motility, which are directly related to the invasion and migration of cancer cells. In summary, lipid metabolic reprogramming, as a common feature in cancer cells, significantly contributes to the maintenance of malignant biological behaviour in cancer cells.

### 2.2. Cancer Cell Pathways Driven by Lipid Metabolites

#### 2.2.1 Regulation of Oncogenic Signalling

In addition to being utilised as substrates for membrane synthesis or energy production, lipids are also important in signal transduction platforms and their metabolites are implicated in several pathways within the TME. In this section, we first discuss the lysophosphatidylcholine (LPC)-autotaxin (ATX)-lysophosphatidic acid (LPA) axis that plays a critical role in tumourigenesis and cancer cell invasion.

**Figure 3:**
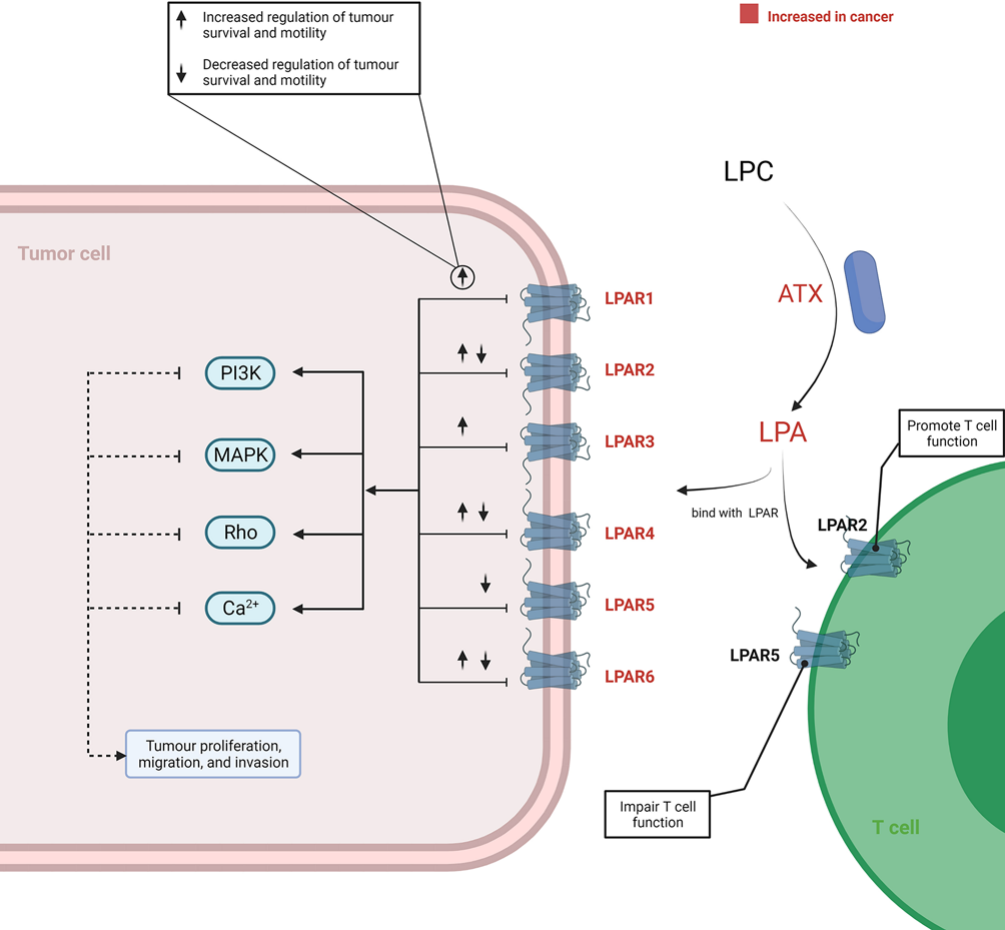
Overview of the LPC/ATX/LPA signalling axis in cancer. LPA is produced *via* ATX-mediated hydrolysis of LPC. LPA-LPAR signalling is magnified and utilised by cancer cells to support their growth and metastasis through increasing the level ATX and LPAR within the TME. When LPA binds with LPAR1-6, it can generate various effects on cancer cells. This process involves the induction of several signalling cascades, including PI3K, MAPK, Rho, and Ca2+ mobilisation-related signalling. T cells under the TME can also be promoted or impaired through binding with different LPARs by LPA. The figure was created with BioRender.com. ATX, autotaxin; LPA, lysophosphatidic acid; LPAR, LPA receptors; LPC, lysophosphatidylcholine; TME, tumour microenvironment.

In the TME, the tumourigenic role of this axis was initiated by ATX, as it served as a plasma lysophospholipase D producing LPA by hydrolysing LPC. Increased expression of ATX was reported in various cancer tissues, including thyroid (45), lung (46), breast (47), hepatic (48), pancreatic (8, 49), renal (50), bladder (50), prostate (51), ovarian (52), and endometrial (53, 54) cancer. The underlying mechanism of an elevated ATX level in cancer has not been completely elucidated. However, *in vitro* studies have reported that an increased ATX was related to the activation of STAT3 in both breast cancer (55) and pancreatic neuroendocrine neoplasms (56). Consequently, the LPC/ATX/LPA axis was overactivated within the TME and subsequently utilised to stimulate tumour progression and metastasis by binding LPA with the G protein-coupled receptor (GPCR) (**Figure 3**). Although a complex network is implicated after the binding of LPA and its receptor, it is well recognised that the pro-tumour process involves activation of the PI3K, MAPK, and Rho signal cascades and Ca2+ mobilisation (57–60). Recently, attention has been paid to the variant roles of different LPA receptors (LPAR) in influencing cancer cell migration, proliferation, and metastasis (61). LPA transfers signals through at least six GPCRs (LPAR1–6) (61–63). The different members of the LPAR family show differentiated effects for cancer cells. In addition, the effects signalled by the same LPAR can also vary across different cancer types. Proliferation and/or motility of cancer cells were promoted after LPA signalling through the LPAR1 in colon, gastric, and breast cancer (64–67), LPAR2 in colon and renal cancer (68–70), LPAR3 in colon, pancreatic, and breast cancer (71–73), LPAR4 in fibrosarcoma (74), and LPAR6 in hepatic and pancreatic cancer (75, 76). Reversely, proliferation and/or motility of cancer cells also showed a decrease after LPA signalling through LPAR2 in melanoma (77), LPAR4 in pancreatic, colon, and head and neck cancer (75, 78, 79), LPAR5 in pancreatic cancer and melanoma (75, 80), and LPAR6 in colon cancer (78, 81). These suggested that the role of the LPC/ATX/LPA axis depends on the types of LPAR and cancer. Thus, there is a need to distinguish the expression patterns and function features of LAPR in further work by applying high-throughput molecular biological techniques aimed at understanding how to improve current therapeutic modalities by targeting the LPC/ATX/LPA axis.

#### 2.2.2 Influence of Immune-Regulated Signalling

Numerous immunotherapy strategies have been developed against cancer, and, among those, inhibition of immune checkpoints holds the greatest promise. The PD-1/PD-L1 axis is one of the most important and well-studied checkpoint pathways in cancer immunity. In recent years, a special role of lipids was revealed in regulation of the PD-1/PD-L1 axis.

PD-1 is a cell surface receptor encoded by the *PDCD1* gene. PD-L1, also known as B7-H1 or CD274, is the ligand of PD-1. The PD-1/PD-L1 axis plays a role in halting or limiting the development of the adaptive immune response (82). It is hijacked by cancer cells to promote T cell exhaustion and the acquisition of immune evasion. PD-L1 is expressed on cancer cells, immune cells, and other TME cells, whereas PD-1 is mainly expressed on T cells and B cells (83, 84). Modification of PD-L1 on cancer cells can greatly influence the immune-regulated function of this axis. Palmitoylation of PD-L1 is now recognised as one of these important modifications. Palmitoylation is a reversible lipid modification of proteins, in which FAs, such as palmitic acid, are attached to a cysteine (S-palmitoylation) residue as a vast majority *via* a thioester linkage (85). This posttranslational modification regulates the localisation and function of proteins (86). Palmitoylation of PD-L1 in cancer cells has demonstrated a contribution to the maintenance of PD-L1 stability and immune evasion of cancer (87, 88). Yang et al. revealed a single palmitoylation site at Cys272 of PD-L1 located in the cytosolic domain, and ZDHHC9 was demonstrated to be responsible for PD-L1 palmitoylation. In breast cells, knockdown of ZDHHC9 decreased PD-L1 palmitoylation following reduction of the PD-L1 protein level and sensitised the T-cell killing function both *in vitro* and *in vivo* (88). These were also demonstrated by Yao et al. (87). Different from the results in breast cancer, they identified that palmitoyltransferase ZDHHC3 was the main acyltransferase for PD-L1 palmitoylation in colorectal cancer cells. Similarly, inhibition of palmitoylation by 2-bromopalmitate and a synthetic peptide successfully induced decrease of PD-L1 expression and enhanced T-cell immunity against tumours *in vitro* and *in vivo* (87). These recent studies indicated a possible strategy to restore the anti-tumour immune response by targeting palmitoylation of PD-L1. As different acyltransferases were identified in different cancer types, further work is needed to verify the unique role of acyltransferases in multiple cancers. In addition, it is also of interest to uncover whether palmitoylation operates similarly in the immune cells under the TME.

## 3. Lipids and Immune Responses in Cancer

Immune cells are an important part of the TME, and they work in tandem to clear cancer cells (anti-tumour response) or to promote cancer progression (immunosuppressive response) by identifying related immune signals. During the anti-tumour response for effective killing of cancer cells, stepwise events have to be initiated and allowed (89). First, neoantigens generated by oncogenesis are released and captured by dendritic cells (DCs) for processing. Then, the captured antigens on MHCI and MHCII molecules were present by DCs to T cells. This results in priming and activation of effector T cell responses against cancer-specific antigens. Finally, effector T cells are recruited and infiltrate into the tumour bed, leading to targeted killing of cancer cells through recognising the cognate antigen bound to MCHI by TCR. However, this process is also characterised by overcoming inhibitory factors that can switch off or limit the anti-tumour response (90). Although the roles of various immune cells in cancer are being revealed, lipids appear to complicate matters. As mentioned in *Interaction Between Lipids and Tumour Cells*, excessive uptake of exogenous lipids and *de novo* synthesis in cancer cells lead to lipid accumulation in the TME, although cancer cells themselves utilise these lipids in various mechanisms (91). To adapt to this increase in lipids, immune cells in the TME have to adjust their metabolic status and the use of lipids, allowing an alteration of their functions. In this chapter, we discussed how lipids interact with various immune cells and change their functions. These impacts on each immune cell are summarised in **Table 1**. Although lipids can have individual consequences on differing or individual immune cell subsets, they mostly act deleteriously against immune cells in their actions against tumours.

**Table 1:** Impact of lipid metabolism and signalling on the tumour immune microenvironment.

Cell types	Lipid abnormality	Impact on cell differentiation and function	Impact on anti-tumour response
**CD8+T cells**	Increased lipid uptake and FAO; triggering the LPC-ATX-LPA axis, PPAR, and tumour-derived PGE2 signalling	Inhibition or enhancement of effector function depending on condition	Unsure
**Tregs**	Increased lipid biosynthesis and FAO; upregulation of CD36; triggering the PPAR signalling	Obtaining metabolic adaptations and advantages to survive and proliferate	Immunosuppressive effects
**Macrophages**	Lipid accumulation and increased FAO; cholesterol efflux	Polarisation to an M2 phenotype	Immunosuppressive effects
**Neutrophils**	Increased FAO	Maintaining ROS production and T cell suppression	Immunosuppressive effects
**MDSC**	Upregulation of FATP2; increased uptake of arachidonic acid and synthesis of PGE2; increased FAO; triggering tumour-derived PGE2 signalling	Promoting T cell suppression by MDSC	Immunosuppressive effects
**DC**	Increased LD accumulation and FAO; triggering tumour-derived PGE2 signalling	Limiting the recruitment of DCs and leading to dysfunction	Immunosuppressive effects
**NK**	Increased uptake and accumulation of exogenous lipids; triggering tumour-derived PGE2 signalling	Impairing cell viability and blunt function	Immunosuppressive effects

ATX, autotaxin; DCs, dendritic cells; FAO, fatty acid oxidation; FATP2, fatty acid transport protein 2; LDs, lipid droplets; LPA, lysophosphatidic acid; LPC, lysophosphatidylcholine; MDSC, myeloid-derived suppressor cells; NK, natural killer; PGE2: prostaglandin-E2; PPAR, peroxisome proliferator-activated receptors; ROS, reactive oxygen species; Tregs, immune regulatory T cells.

### 3.1. T Cells

CD8+ and CD4+ T cells are two important lymphocyte subtypes in the TME. CD8+T cells, also known as CTL, can kill and eliminate cancer cells by releasing perforin, granzymes, and other effector molecules. CD4+T cells include T-helper 1 (Th1) cells, T-helper 2 (Th2) cells, T-helper 17 (Th17) cells, and immune regulatory T cells (Tregs). Tregs are the cell types most commonly associated with immunosuppression among the CD4+T cell subtypes.

The effector function of CD8+T cells is predominantly maintained by aerobic glycolysis. However, if both oxygen and glucose are lacking in the TME, CD8+T cells will increase FA uptake and catabolism (92), and FAO may be initiated and utilised. Regarding the impact of lipid metabolism on CD8+T cells, conflicting results have been reported across different studies. Some studies have shown an immunosuppressive effect of lipid metabolism in CD8+T cells. The immune checkpoint, PD-1, was found to mediate the blockade of T-effector cell differentiation by inhibiting glycolysis and promoting FAO (93). In breast cancer, obesity-driven leptin/STAT3 signalling promoted FAO and reduced glycolysis in CD8+ T-effector cells, consequently leading to the inhibition of effector functions and facilitation of tumour growth (94). It was also reported by experimental studies that pretreatment with tumour-derived free FA can significantly reduce the effector activity of CD8+ cells (95, 96). In contrast, mouse model studies by Chowdhury et al. (97) and Saibil et al. (98) showed that FAO in T cells can be increased *via* upregulation of carnitine palmitoyl transferase 1a (CPT1a, the rate-limiting enzyme of FAO) induced by peroxisome proliferator-activated receptor (PPAR) activation, and an increase of FAO promoted proliferation of CD8+ T cells, reduced their apoptosis, and enhanced the effector function against tumours; however, it was noted that this may not be fully attributable to FAO, as glycolysis is also upregulated (97). Similar contradictions can also be found in the role of cholesterol in CD8+ T cell function. The cholesterol level in CD8+T cells contributes to its exhaustion by upregulating immune checkpoints induced by endoplasmic reticulum stress (99). However, another study assessed the association between the cholesterol level in membranes and the effector function of CD8+T cells and showed that increasing the cholesterol level in the membranes of CD8+T cells by inhibiting cholesterol esterification caused an enhanced anti-tumour effect in melanoma (100). Lipid metabolites, as signalling molecules, play multiple roles in adjusting T cell effector function. The LPC-ATX-LPA axis is intimately implicated in lipid-related signalling. Signals of LPA through LPAR2 have been reported to have pro-T cell function, which suggests that LPA signalling may improve the immune response against tumours (101). However, LPA signals through LPAR5 in T cells are associated with impaired T cell cytotoxicity (102, 103). The prostaglandin-mediated signalling pathway has also been examined. Prostaglandins are a group of lipid compounds that are enzymatically derived from arachidonic acid. It was found that overproduction of prostaglandin-E2 (PGE2) by metastatic murine renal carcinoma (Renca) cells blocked the priming of tumour-special CTLs *in vivo* by abrogating IFNγ-dependent costimulatory signalling between ICAM-1 and the lymphocyte receptor LFA-1 (104). In summary, the effects of lipids on CD8+T cells are multiple, and the roles of facilitating or inhibiting CD8+ T cell effector functions may depend on the categories, location, and sources of lipids and patterns of cross talk and reactions between lipids and the CD8+ T cells.

Tregs express FoxP3, a regulatory molecule that enhances Treg resistance to lipotoxic environments (105). Within the TME, Tregs have enhanced glycolytic rates and lipid biosynthesis and rely on FAO for expansion (106, 107). Compared to CD8+T cells, Tregs have critical advantages for survival and proliferation in the absence of oxygen or glucose because of their special metabolism profile, including increased FAO (12, 108). In this process, PPAR signalling plays a crucial role in mediating the metabolic adaptation of Tregs in the TME. It was demonstrated that intratumoural Tregs from patients with cancer or mouse models highly expressed CD36 (109). Further experiments revealed that CD36 promoted mitochondrial fitness in intratumoural Treg cells *via* enhancing lipid uptake and activating the PPAR-β pathways, leading to support for Treg persistence in the TME and maintenance of their suppressive function. With the help of the aforementioned metabolic adaptation, Tregs within the TME play an important role in the immune evasion of tumour cells. Modulation of Treg number and function through disrupting their lipids metabolism may be a viable strategy to elicit the anti-tumour response and enhance immunotherapy.

### 3.2. Macrophages

Macrophages account for the largest fraction of immune cells in some cancers and exhibit phenotypic plasticity under different conditions (110). According to their functional characteristics, surface markers, and secreted cytokines, macrophages are usually classified into two types: M1 (pro-inflammatory) and M2 (anti-inflammatory), although many new subclasses of macrophages are now being reported. In the TME, tumour-associated macrophages (TAMs) generally polarise to the M2 phenotype and enable the immune evasion of cancer cells by suppressing T cell activation and inducing Treg recruitment (111).

M2-like TAMs are prone to increase FAO because the TME is an FA-rich environment (112, 113). Several studies have determined that lipid accumulation and FAO are important in maintaining the immunosuppressive phenotype of TAMs (11, 114, 115). Cholesterol metabolism can also influence TAM function. It has been reported in a mouse model of metastatic ovarian cancer that cholesterol efflux in TAMs supports IL-4 signalling and promotes tumour progression (116). Besides, PGE2-mediated signalling may contribute to the M2 polarisation of macrophages. Wang et al. reported that treating macrophages with PGE2, a lipid mediator that can be highly produced by cancer, inhibited the expression of miR-21 that was a helpful microRNA for polarisation of macrophages to M1 types (117). However, the types of lipids or metabolic mechanisms that are critical for TAM differentiation and polarisation remain poorly understood (118–125). Further studies are expected to clarify the complex cross talk between macrophages and lipids.

### 3.3. Neutrophils and Myeloid-Derived Suppressor Cells

Neutrophils are the most abundant type of granulocytes. Neutrophils in the TME generally facilitate tumour progression, during which metabolism plays an important role. Since the TME has low glucose but high lipid levels, neutrophils can utilise FAO to maintain immune suppression (126), consequently leading to tumour progression.

Myeloid-derived suppressor cells (MDSCs) are a heterogeneous population of pathologically activated myeloid precursors and relatively immature myeloid cells (127, 128). They can be divided into two types: polymorphonuclear (PMN)—MDSCs and monocytic (M)—MDSCs. PMN-MDSCs account for most MDSC populations in humans and mice. PMN-MDSCs are similar to neutrophils in many morphological and phenotypic features. In the TME, PMN-MDSCs prefer FAO as a primary source of energy and act as immunosuppressive cells (129). FAO may help in the T cell suppression of PMC-MDSCs through ROS-peroxynitrite generation (130). It was recently demonstrated that FA metabolites contribute to PMN-MDSC-mediated immunosuppression (10). FA transport protein 2 (FATP2, encoded by the Slc27a2 gene) was found to be upregulated exclusively in PMN-MDSCs of tumour-bearing mice and increased lipid accumulation in PMN-MDSCs. Knocking out of FATP2 abrogated the suppression of CD8+ T cells by PMN-MDSCs, thereby delaying tumour growth (10). It was further demonstrated that FATP2-mediated immune suppression was achieved through increasing the uptake of arachidonic acid and the synthesis of PGE2 in PMN-MDSCs (10). In another study, it was also found that tumour-derived PGE2 greatly contributed to the activation of MDSCs (131). This study demonstrated in both *in vitro* and *in vivo* models that drug-resistant breast cancer cells used secreted PGE2 to promote MDSC expansion and polarisation by upregulation of miR-10 and consequent triggering of AMPK signalling.

### 3.4. Others

Other important immune cells involved in anti-tumour immunity include dendritic cells (DCs) and natural killer (NK) cells. DCs are the most powerful antigen-presenting cells and play an important role in the activation of T cells. NK cells are important cells in the innate immune system. In the TME, NK cells induce cancer cell death by releasing perforin and granzymes or through death receptor-ligand engagement. NK cells can also regulate the function of other immune cells by secreting cytokines and chemokines. Lipid metabolism has a significant influence on the activation and function of both DC and NK cells.

DCs increase glycolysis and FA synthesis after activation by toll-like receptor stimulation (132). Increased lipid deposition was observed in tumour-associated DCs, and this alteration of the lipid level was caused by increased lipid uptake due to upregulation of class A macrophage scavenger receptor type 1 (Msr1) (133). The accumulation of lipid droplets (LDs) in tumour-associated DCs contributes to DC dysfunction by reducing antigen presentation and attenuating T cell activation (133–137). In addition to lipid storage in DCs, FAO was considered to have an active role in driving DCs towards a tolerogenic phenotype (138) possibly because the increased lipid storages in tumour-associated DCs can serve to fuel the FAO process by the stimulation of PPAR signalling (139). NK cells can be negatively affected by exogenous lipids, especially in the context of obesity (140–142). It was reported that obesity was associated with robust PPAR-driven lipid uptake and accumulation in NK cells (13). The lipid accumulation forced NK cells to express more lipids transporters and enzymes involved in FAO to avoid lipotoxicity, which could limit the mTOR-mediated glycolysis increase (13). As mTOR-dependent metabolic reprogramming is a prerequisite for NK cell effector function; it led to loss of cytotoxicity production by NK cells, such as granzyme B and IFN-γ, and a consequent failure to attack tumours under conditions of obesity (13, 143).

In addition, tumour-derived prostaglandin signalling also has an effect on both these cells. Park et al. showed that thyroid cancer-derived PGE2 suppressed NK cell maturation and their cytolytic activity by inhibiting the expression of NK receptors, such as NK44, NK30, TRAIL, and NKG2D (144). Inhibition of PGE2 signalling by targeting the PGE2 receptor EP4 could restore the activation of NK cells in tumour-bearing mice (145). Another study by Böttcher et al. revealed an interaction between NK cells and DCs (146). They found in a mouse model that the accumulation of conventional type 1 dendritic cells (cDC1) in the TME relies on the chemoattractants CCL5 and XCL1 produced by NK cells. Tumour-derived PGE2 impairs NK cell viability and chemokine production and downregulates chemokine receptor expression in cDC1, consequently resulting in the evasion of the anti-tumour immune response (146).

## 4. Lipids and Their Potential Applications

**Figure 4:**
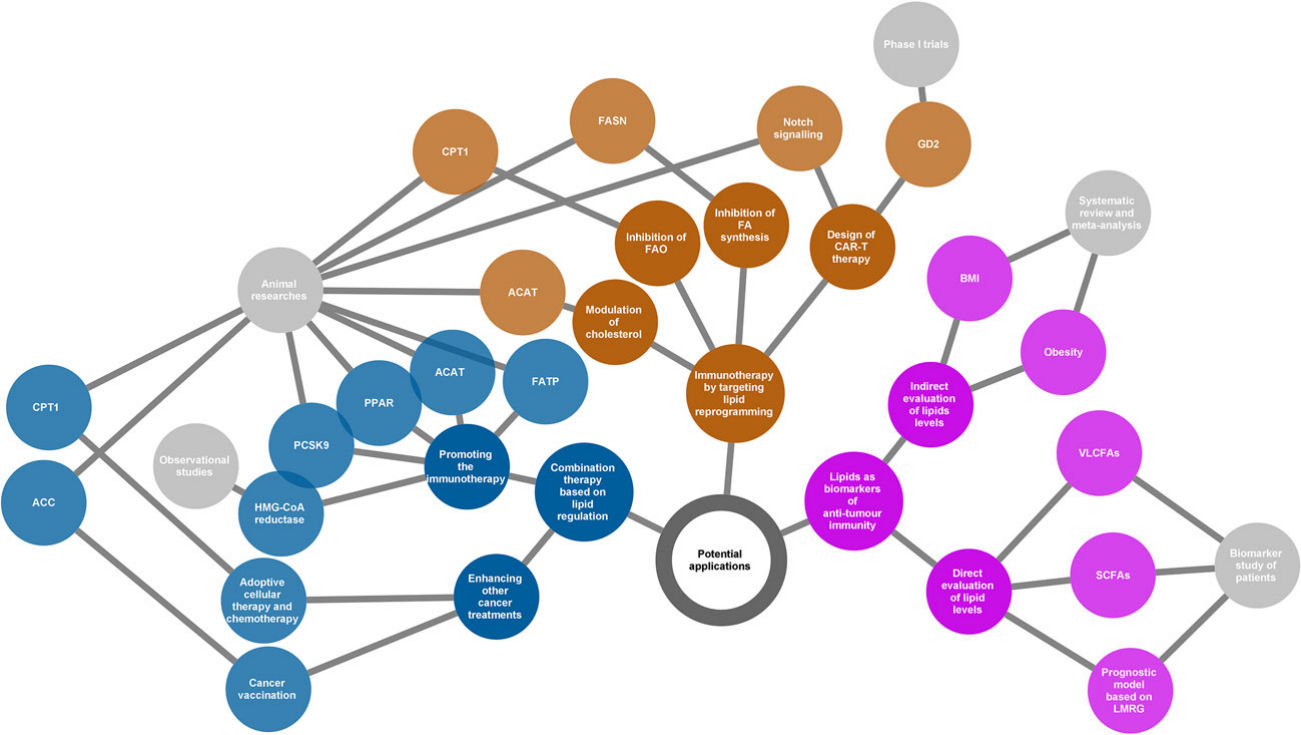
Potential applications of the lipids according to their various roles in cancer immunity. Three directions presented (yellow, blue, and purple nodes) for potential applications as summarized in the network diagram. The grey nodes in the last level indicate evidence types supporting each application. The adjacently spread nodes next to the last nodes represent lipids-related therapeutic targets or biomarkers. ACC, acetyl-CoA carboxylase; ACAT, acetyl-CoA acetyltransferase; BMI, body mass index; CAR-T, chimeric antigen receptor T cell; CPT1, carnitine palmitoyltransferase 1; FA, fatty acid; FAO, fatty acid oxidation; FASN, fatty acid synthase; FATP, transport protein; GD2, a disialoganglioside glycolipid; LMRG, lipid metabolism-related genes; PCSK9, proprotein convertase subtilisin/kexin type 9; PPAR, peroxisome proliferator-activated receptor; SCFAs, short-chain fatty acids; VLCFAs, very long-chain fatty acids.

A continuous in-depth understanding of the contribution of lipid metabolism and signalling to cancer immunity will provide insight into the clinical translation of lipid substances. It may be difficult to acquire the desired results based on a single strategy because of the complex interaction between lipids and cancer immunity. However, lipids have shown tremendous potential for development as biomarkers and therapeutic targets (**Figure 4**). In this chapter, we review the current potential directions of lipid application based on their cross talk with cancer immunity and highlight what has been achieved and what should be overcome in the future.

### 4.1. New Immunotherapeutic Strategies by Targeting Lipid Reprogramming

Chimeric antigen receptor T cell (CAR-T) therapy is a promising new approach to fight cancer using T cells engineered with CARs that provide these cells with the ability to target specific antigens on the cancer cell surface. CAR-T therapy has been demonstrated to be successful in the treatment of haematologic cancers (147, 148); however, its application in solid tumours is still challenging (149). GD2, a disialoganglioside glycolipid, is a promising target in solid tumour CAR-T therapy. GD2 is normally present in peripheral neurons and parts of the central nervous system but can be overexpressed in some tumours, such as neuroblastoma and melanoma (150). Several phase I trials have demonstrated that GD2-specific CAR T cell therapy is safe and effective for treating neuroblastoma (151–153). The major challenge of CAR-T therapy is the difficulty in maintaining strong and persistent tumour remission. A novel strategy that is based on targeting lipid metabolism has been designed to address this problem. As Notch signalling can promote mitochondrial biogenesis and FA synthesis, a recent study showed that activation of Notch and overexpression of its downstream gene FOXM1 converted conventional human CAR-T cells into T_SCM_(stem cell memory T)-like CAR-T cells and enhanced the anti-tumour effects of CAR-T therapy in an *in vivo* model of leukaemia (154).

An increase in lipid metabolism in the TME leads to intracellular lipid accumulation and promotes FAO in immune cells. The immunosuppressive phenotypes generally rely on FAO for energy support. Thus, changing the suppressive phenotypes to responsive phenotypes by ameliorating lipid abundance is a theoretically practical approach to attacking cancer. Anti-tumour strategies targeting both FA synthesis and FAO are beneficial. Inhibition of FASN, the key metabolic enzyme of *de novo* lipogenesis, using its small molecule inhibitor cerulenin, partly restored the function of DC cells and resulted in extended tumour control in a mouse model of ovarian cancer (155). According to another study on FA metabolism and apoptosis sensitivity of human T cells, inhibition of FASN by treatment with a compound C75 could protect T cells in the TME, caused by repeated TCR activation, from apoptosis (156). This can also enhance anti-tumour immunity in addition to directly affecting the function of cancer cells. Alternatively, pharmacological inhibition of FAO using the inhibitor of CPT1, the rate-limiting enzyme in FAO cycle, can restore the function of tumour effector T cells but block the production of inhibitory cytokines from MDSC, consequently leading to a delay of tumour growth *in vivo* in tumour-bearing mice (94, 130). In addition, cholesterol modulation is a new strategy for cancer immunotherapy. A study showed that inhibition of ACAT1, a key cholesterol esterification enzyme, significantly enhanced the effector function of CD8+ T cells by increasing the plasma membrane cholesterol level. Avasimibe, a small-molecule inhibitor of ACAT, was used to treat melanoma in mice and was observed to have a beneficial anti-tumour effect (100).

In summary, targeting lipid reprogramming shows great promise in the treatment of cancer with great potential to bring cancer immunotherapy into a new era. However, the evidence of these new immunotherapeutic strategies is still based on results from preclinical and phase I trials. In addition, it is also necessary to note that targeting lipid metabolism may affect multiple immune populations, and thus the linked outcomes could be unpredictable. The translational potential and clinical significance of this strategy warrants further study.

### 4.2. Combination Therapy Based on Lipid Regulation

Beyond playing lead roles in cancer immunotherapy, lipid regulation can be used as an adjuvant to other therapeutic measures such as radiotherapy, chemotherapy, and immune checkpoint inhibitors. This is based on the fact that lipid modification will help to improve the TME.

Bezafibrate, an agonist of PPAR, has a proven synergistic effect with a PD-1-blocking monoclonal antibody in mice model (97, 157). The underlying mechanism was a sustained increase in the number of functional CD8 + T cells through inducing intracellular FAO. Inhibition of FA transport also enhanced the therapeutic benefit of several immunomodulatory therapies, including anti-PD-1, anti-CTLA-4, and anti-CSF-1R treatments (10). In addition, combined treatment of cholesterol metabolism-targeted drugs and immunotherapy has also been widely evaluated in both preclinical and clinical studies. For example, preclinical data showed that inhibition of PCSK9 (proprotein convertase subtilisin/kexin type 9) (158) or ACAT1 (Acetyl-CoA acetyltransferase 1) (100) can improve the anti-tumour effect of the anti-PD-1 antibody in multiple cancers. Similarly, clinical data confirmed an increased objective response rate and improved survival when combined with statins in PD-1/PD-L1 checkpoint inhibitors-treated patients (159–161). Apart from the combined strategy for immunotherapy, there is also evidence regarding the enhanced anti-tumour effects of lipid modulation by activated immune function in other cancer treatments. For example, using TOFA, an ACC inhibitor that participates in FAS, normalised lipid levels in DCs. As a result, TOFA restored the activity of DCs and enhanced the anti-tumour potency of cancer vaccination in both tumour-bearing mice of melanoma and lymphoma (133). Etomoxir, an inhibitor of CPT1, in combination with adoptive cellular therapy, showed a significantly better anti-tumour effect *in vivo* compared to adoptive cellular therapy alone. This benefit was related to the increased infiltration of adoptively transferred T cells in the TME and increased production of IFN-γ (130). Similarly, FAO inhibition by etomoxir can significantly increase the anti-tumour effect of chemotherapy *in vivo* by targeting MDSC-associated immunosuppressive effects (130).

Based on the understanding of the association between lipid reprogramming and immune cell phenotypes, lipid modulation could offer new opportunities for strengthening cancer therapy. Although substantial evidence has demonstrated the enormous capacity of the reprogramming of lipid metabolism in improving the tumour response, the functional mechanism of combination treatment may be complicated, and much remains to be done before it can be applied to clinical practice. Most current studies have used preclinical models or were based on retrospective populations, and clinical-trial evidence is lacking. Therefore, prospective trials are needed to verify the effectiveness of lipid regulation in cancer treatment under real-world conditions.

### 4.3. Lipids as Biomarkers of Anti-Tumour Immunity

The cross talk between lipids and cancer immunity provides us with an opportunity to use lipids as biomarkers in evaluating the immune response of cancer. This possibility relies on the development of technologies for the qualitative and quantitative analyses of metabolites, such as next-generation sequencing and liquid biopsy. Based on the understanding of the immune profile of cancer, lipids have been identified as predictors of discrimination prognosis, immune phenotypes, and treatment response.

Immune checkpoint inhibitors have shown great success because of their powerful anti-tumour ability; however, only a small population can benefit from immunotherapy. Thus, it is of great interest to discover potential biomarkers that can predict the response to immune checkpoint inhibitors. A recent study observed that serum concentrations of very long-chain FAs (VLCFAs) could predict the treatment response in patients with urological cancer treated with immune checkpoint inhibitors (nivolumab or atezolizumab) (162). The rationale for this biomarker may be related to lipid metabolism in immune cells. At present, emerging evidence has shown that the gut microbiota is closely related to the response to immunotherapy (163–166). Short-chain FAs (SCFAs) such as acetate, propionate, and butyrate are the major end products derived from the gut microbiota. Studies have shown that high concentrations of SCFAs are significantly associated with better outcomes in patients receiving anti-PD-1 therapy (167, 168). The ability of SCFAs to regulate immune function may explain the prediction of treatment outcomes. Obesity or body mass index (BMI), as a surrogate of lipid levels, was also identified as a predictor of immunotherapy. Several studies have determined that a high BMI or obesity was predictive of improved overall survival or progression-free survival in patients with cancer treated with immune checkpoint inhibitors (169, 170). In addition, some bioinformatics analyses based on ovarian cancer and gliomas indicated that lipid metabolism-related genes could be used as prognostic factors and are associated with the immune phenotypes of patients with cancer (171, 172).

Although several lipid molecules or derivatives have been identified as good biomarkers of the anti-tumour response, the interaction between a characteristic lipid profile and cancer immunity is still poorly understood. With an increasing number of lipid-related components being explored to identify immune responses to cancer, we should pay more attention to the availability of their predictive ability in a wider population based on the complicated roles of lipids in immune regulation.

## 5. Conclusions

Remodelling of cancer immunity is now demonstrated to be an effective approach to treat cancer. A systematic understanding of the variables that disturb the immune response to cancer will help us overcome the limitations of current immunotherapeutic strategies. Substantial evidence indicates that lipids have an important role in cancer immunity. Both lipid uptake and *de novo* lipogenesis are increased in cancer cells, providing materials and energy for cell proliferation. Cancer cells can also exhibit aggressive behaviour to avoid elimination by the immune system through lipid metabolic reprogramming. In addition, biomolecules from lipid metabolism contribute to the transduction of immune inhibition and oncogenic signals in cancer cells. Conversely, lipids within the TME are involved in cancer immunity by affecting the survival, differentiation, and action of immune cells. The results of disturbance by lipids depend on the condition of the TME, but generally lead to an immunosuppressive effect. Determining how lipid metabolism impacts cancer immunity and mediates crosstalk between different cells can provide insight into treatment strategies against cancer. Targeting lipid reprogramming may create a new path in the field of immunotherapy. Another easy strategy is to strengthen other treatment measures by activating anti-tumour immunity through lipid regulation. Based on the close relationship between lipids and cancer immunity, lipids can also be used as biomarkers for the selection of advantaged populations, surveillance of immune conditions, and evaluation of treatment response. However, the complicated roles of lipids in the TME may lead to unexpected outcomes when translating them into the clinical environment. Continuing to unravel the interaction between lipids and cancer immunity will bring cancer management into a new era. 

## Author Contributions

Conceptualisation, YY and HC. Writing—original draft preparation, YY, LG, YW, and BX. Visualisation, YY. Writing—review and editing, EM, HL, PZ, PT, LX, BG, AL, and HC. All authors have read and agreed to the published version of the manuscript.

## Funding

This research was funded by the Key Talents Project of Gansu Province (grant no. 2019RCXM020), Key Project of Science and Technology in Gansu province (grant no. 19ZD2WA001), Science and Technology Project of Chengguan District of Lanzhou City (grant nos. 2019RCCX0034, 2020SHFZ0039 and 2020JSCX0073), and Cuiying Scientific and Technological Innovation Program of Lanzhou University Second Hospital (grant no. CY2017-ZD01).

## Conflict of Interest

The authors declare that the research was conducted in the absence of any commercial or financial relationships that could be construed as a potential conflict of interest.

## Publisher’s Note

All claims expressed in this article are solely those of the authors and do not necessarily represent those of their affiliated organizations, or those of the publisher, the editors and the reviewers. Any product that may be evaluated in this article, or claim that may be made by its manufacturer, is not guaranteed or endorsed by the publisher.

## Abbreviations

ACAT1, acetyl-CoA acetyltransferase 1; ACC, acetyl-CoA carboxylase; ACLY, ATP citrate lyase; ATX, autotaxin; BMI, body mass index; CAR-T, chimeric antigen receptor T cell; cDC1, conventional type 1 dendritic cells; CPT1a, carnitine palmitoyl transferase 1a; DCs, dendritic cells; FABPs, fatty acid-binding proteins; FAO, fatty acid oxidation; FAs, fatty acids; FASN, fatty acid synthase; FATPs, fatty acid transport proteins; FATP2, FATP 2; GPCR: G protein-coupled receptor receptors; LDs, lipid droplets; LDLR, low-density lipoprotein receptor; LPA, lysophosphatidic acid; LPAR: LPA receptors; LPC, lysophosphatidylcholine; MDSCs, myeloid-derived suppressor cells; M-MDSCs, monocytic-MDSCs; Msr1: macrophage scavenger receptor type 1; NK cells, natural killer cells; PGE2, prostaglandin-E2; PCSK9: proprotein convertase subtilisin/kexin type 9; PMN-MDSCs, polymorphonuclear-MDSCs; PPAR, peroxisome proliferator-activated receptor; PUFA: polyunsaturated fatty acid; SCD, stearoyl-CoA desaturase (Δ9); SCFAs, short-chain fatty acids; SREBPs: sterol regulatory element-binding proteins; TAM, tumour-associated macrophages; Th1, T-helper 1; Th17, T-helper 17; Th2, T-helper 2; TME, tumour microenvironment; Tregs, immune regulatory T cells; T_SCM_, stem cell memory T; VLCFAs, very long-chain fatty acids; VLDL, very low-density lipoproteins.

## References

[1] Boyce JA. Eicosanoids in Asthma, Allergic Inflammation, and Host Defense. Curr Mol Med (2008) 8:335–49. doi: 10.2174/156652408785160989 18691060

[2] Eyster KM. The Membrane and Lipids as Integral Participants in Signal Transduction: Lipid Signal Transduction for the Non-Lipid Biochemist. Adv Physiol Education (2007) 31:5–16. doi: 10.1152/advan.00088.2006 17327576

[3] Hinkovska-Galcheva V VanWay SM Shanley TP Kunkel RG. The Role of Sphingosine-1-Phosphate and Ceramide-1-Phosphate in Calcium Homeostasis. Curr Opin Investigational Drugs (London England: 2000) (2008) 9:1192–205. 18951299

[4] Wen YA Xing X Harris JW Zaytseva YY Mitov MI Napier DL. Adipocytes Activate Mitochondrial Fatty Acid Oxidation and Autophagy to Promote Tumor Growth in Colon Cancer. Cell Death Disease (2017) 8:e2593. doi: 10.1038/cddis.2017.21 28151470 PMC5386470

[5] Lin H Patel S Affleck VS Wilson I Turnbull DM Joshi AR. Fatty Acid Oxidation Is Required for the Respiration and Proliferation of Malignant Glioma Cells. Neuro-Oncology (2017) 19:43–54. doi: 10.1093/neuonc/now128 27365097 PMC5193020

[6] Camarda R Zhou AY Kohnz RA Balakrishnan S Mahieu C Anderton B. Inhibition of Fatty Acid Oxidation as a Therapy for MYC-Overexpressing Triple-Negative Breast Cancer. Nat Med (2016) 22:427–32. doi: 10.1038/nm.4055 PMC4892846 26950360

[7] Volden PA Skor MN Johnson MB Singh P Patel FN McClintock MK. Mammary Adipose Tissue-Derived Lysophospholipids Promote Estrogen Receptor-Negative Mammary Epithelial Cell Proliferation. Cancer Prev Res (Philadelphia Pa.) (2016) 9:367–78. doi: 10.1158/1940-6207.Capr-15-0107 PMC4854771 26862086

[8] Auciello FR Bulusu V Oon C Tait-Mulder J Berry M Bhattacharyya S. A Stromal Lysolipid-Autotaxin Signaling Axis Promotes Pancreatic Tumor Progression. Cancer Discov (2019) 9:617–27. doi: 10.1158/2159-8290.Cd-18-1212 PMC6497553 30837243

[9] Benesch MG Tang X Dewald J Dong WF Mackey JR Hemmings DG. Tumor-Induced Inflammation in Mammary Adipose Tissue Stimulates a Vicious Cycle of Autotaxin Expression and Breast Cancer Progression. FASEB J Off Publ Fed Am Societies Exp Biol (2015) 29:3990–4000. doi: 10.1096/fj.15-274480 26071407

[10] Veglia F Tyurin VA Blasi M De Leo A Kossenkov AV Donthireddy L. Fatty Acid Transport Protein 2 Reprograms Neutrophils in Cancer. Nature (2019) 569:73–8. doi: 10.1038/s41586-019-1118-2 PMC6557120 30996346

[11] Wu H Han Y Rodriguez Sillke Y Deng H Siddiqui S Treese C. Lipid Droplet-Dependent Fatty Acid Metabolism Controls the Immune Suppressive Phenotype of Tumor-Associated Macrophages. EMBO Mol Med (2019) 11:e10698. doi: 10.15252/emmm.201910698 31602788 PMC6835560

[12] Miska J Lee-Chang C Rashidi A Muroski ME Chang AL Lopez-Rosas A. HIF-1α Is a Metabolic Switch Between Glycolytic-Driven Migration and Oxidative Phosphorylation-Driven Immunosuppression of Tregs in Glioblastoma. Cell Rep (2019) 27:226–237.e4. doi: 10.1016/j.celrep.2019.03.029 30943404 PMC6461402

[13] Michelet X Dyck L Hogan A Loftus RM Duquette D Wei K. Metabolic Reprogramming of Natural Killer Cells in Obesity Limits Antitumor Responses. Nat Immunol (2018) 19:1330–40. doi: 10.1038/s41590-018-0251-7 30420624

[14] Silverstein RL Febbraio M. CD36, a Scavenger Receptor Involved in Immunity, Metabolism, Angiogenesis, and Behavior. Sci Signaling (2009) 2:re3. doi: 10.1126/scisignal.272re3 PMC2811062 19471024

[15] Yoshida T Yokobori T Saito H Kuriyama K Kumakura Y Honjo H. CD36 Expression Is Associated With Cancer Aggressiveness and Energy Source in Esophageal Squamous Cell Carcinoma. Ann Surg Oncol (2021) 28:1217–27. doi: 10.1245/s10434-020-08711-3 32529269

[16] Pan J Fan Z Wang Z Dai Q Xiang Z Yuan F. CD36 Mediates Palmitate Acid-Induced Metastasis of Gastric Cancer *via* AKT/GSK-3β/β-Catenin Pathway. J Exp Clin Cancer Res CR (2019) 38:52. doi: 10.1186/s13046-019-1049-7 30717785 PMC6360779

[17] Deng M Cai X Long L Xie L Ma H Zhou Y. CD36 Promotes the Epithelial-Mesenchymal Transition and Metastasis in Cervical Cancer by Interacting With TGF-β. J Trans Med (2019) 17:352. doi: 10.1186/s12967-019-2098-6 PMC6815430 31655604

[18] Feng WW Wilkins O Bang S Ung M Li J An J. CD36-Mediated Metabolic Rewiring of Breast Cancer Cells Promotes Resistance to HER2-Targeted Therapies. Cell Rep (2019) 29:3405–3420.e5. doi: 10.1016/j.celrep.2019.11.008 31825825 PMC6938262

[19] Xu WH Qu YY Wang J Wang HK Wan FN Zhao JY. Elevated CD36 Expression Correlates With Increased Visceral Adipose Tissue and Predicts Poor Prognosis in ccRCC Patients. J Cancer (2019) 10:4522–31. doi: 10.7150/jca.30989 PMC6746135 31528216

[20] Zhang M Di Martino JS Bowman RL Campbell NR Baksh SC Simon-Vermot T. Adipocyte-Derived Lipids Mediate Melanoma Progression *via* FATP Proteins. Cancer Discov (2018) 8:1006–25. doi: 10.1158/2159-8290.CD-17-1371 PMC6192670 29903879

[21] Liu RZ Choi WS Jain S Dinakaran D Xu X Han WH. The Fabp12/Pparγ Pathway Promotes Metastatic Transformation by Inducing Epithelial-to-Mesenchymal Transition and Lipid-Derived Energy Production in Prostate Cancer Cells. Mol Oncol (2020) 14:3100. doi: 10.1002/1878-0261.12818 33031638 PMC7718947

[22] Balaban S Shearer RF Lee LS van Geldermalsen M Schreuder M Shtein HC. Adipocyte Lipolysis Links Obesity to Breast Cancer Growth: Adipocyte-Derived Fatty Acids Drive Breast Cancer Cell Proliferation and Migration. Cancer Metab (2017) 5:1. doi: 10.1186/s40170-016-0163-7 28101337 PMC5237166

[23] Dirat B Bochet L Dabek M Daviaud D Dauvillier S Majed B. Cancer-Associated Adipocytes Exhibit an Activated Phenotype and Contribute to Breast Cancer Invasion. Cancer Res (2011) 71:2455–65. doi: 10.1158/0008-5472.Can-10-3323 21459803

[24] Fujisaki K Fujimoto H Sangai T Nagashima T Sakakibara M Shiina N. Cancer-Mediated Adipose Reversion Promotes Cancer Cell Migration *via* IL-6 and MCP-1. Breast Cancer Res Treat (2015) 150:255–63. doi: 10.1007/s10549-015-3318-2 25721605

[25] Wueest S Konrad D. The Role of Adipocyte-Specific IL-6-Type Cytokine Signaling in FFA and Leptin Release. Adipocyte (2018) 7:226–8. doi: 10.1080/21623945.2018.1493901 PMC6224188 30001663

[26] Petruzzelli M Schweiger M Schreiber R Campos-Olivas R Tsoli M Allen J. A Switch From White to Brown Fat Increases Energy Expenditure in Cancer-Associated Cachexia. Cell Metab (2014) 20:433–47. doi: 10.1016/j.cmet.2014.06.011 25043816

[27] Su T Huang C Yang C Jiang T Su J Chen M. Apigenin Inhibits STAT3/CD36 Signaling Axis and Reduces Visceral Obesity. Pharmacol Res (2020) 152:104586. doi: 10.1016/j.phrs.2019.104586 31877350

[28] Rozovski U Harris DM Li P Liu Z Jain P Ferrajoli A. STAT3-Activated CD36 Facilitates Fatty Acid Uptake in Chronic Lymphocytic Leukemia Cells. Oncotarget (2018) 9:21268–80. doi: 10.18632/oncotarget.25066 PMC5940394 29765537

[29] Cao D Song X Che L Li X Pilo MG Vidili G. Both *De Novo* Synthetized and Exogenous Fatty Acids Support the Growth of Hepatocellular Carcinoma Cells. Liver Int Off J Int Assoc Study Liver (2017) 37:80–9. doi: 10.1111/liv.13183 PMC5140766 27264722

[30] Cerne D Melkic E Trost Z Sok M Marc J. Lipoprotein Lipase Activity and Gene Expression in Lung Cancer and in Adjacent Noncancer Lung Tissue. Exp Lung Res (2007) 33:217–25. doi: 10.1080/01902140701481054 17620184

[31] Dong W Gong H Zhang G Vuletic S Albers J Zhang J. Lipoprotein Lipase and Phospholipid Transfer Protein Overexpression in Human Glioma Cells and Their Effect on Cell Growth, Apoptosis, and Migration. Acta Biochim Biophys Sinica (2017) 49:62–73. doi: 10.1093/abbs/gmw117 27864281

[32] Kuemmerle NB Rysman E Lombardo PS Flanagan AJ Lipe BC Wells WA. Lipoprotein Lipase Links Dietary Fat to Solid Tumor Cell Proliferation. Mol Cancer Ther (2011) 10:427–36. doi: 10.1158/1535-7163.Mct-10-0802 PMC3074101 21282354

[33] Lupien LE Bloch K Dehairs J Traphagen NA Feng WW Davis WL. Endocytosis of Very Low-Density Lipoproteins: An Unexpected Mechanism for Lipid Acquisition by Breast Cancer Cells. J Lipid Res (2020) 61:205–18. doi: 10.1194/jlr.RA119000327 PMC6997602 31806729

[34] Koundouros N Poulogiannis G. Reprogramming of Fatty Acid Metabolism in Cancer. Br J Cancer (2020) 122:4–22. doi: 10.1038/s41416-019-0650-z 31819192 PMC6964678

[35] Menendez JA Lupu R. Fatty Acid Synthase and the Lipogenic Phenotype in Cancer Pathogenesis. Nat Rev Cancer (2007) 7:763–77. doi: 10.1038/nrc2222 17882277

[36] Cheng C Geng F Cheng X Guo D. Lipid Metabolism Reprogramming and its Potential Targets in Cancer. Cancer Commun (2018) 38:1–14. doi: 10.1186/s40880-018-0301-4 PMC5993136 29784041

[37] Schiliro C Firestein BL. Mechanisms of Metabolic Reprogramming in Cancer Cells Supporting Enhanced Growth and Proliferation. Cells (2021) 10:1056. doi: 10.3390/cells10051056 33946927 PMC8146072

[38] Yao C-H Fowle-Grider R Mahieu NG Liu G-Y Chen Y-J Wang R. Exogenous Fatty Acids Are the Preferred Source of Membrane Lipids in Proliferating Fibroblasts. Cell Chem Biol (2016) 23:483–93. doi: 10.1016/j.chembiol.2016.03.007 PMC5510604 27049668

[39] Hopperton KE Duncan RE Bazinet RP Archer MC. Fatty Acid Synthase Plays a Role in Cancer Metabolism Beyond Providing Fatty Acids for Phospholipid Synthesis or Sustaining Elevations in Glycolytic Activity. Exp Cell Res (2014) 320:302–10. doi: 10.1016/j.yexcr.2013.10.016 24200503

[40] Hilvo M Denkert C Lehtinen L Müller B Brockmöller S Seppänen-Laakso T. Novel Theranostic Opportunities Offered by Characterization of Altered Membrane Lipid Metabolism in Breast Cancer Progression. Cancer Res (2011) 71:3236–45. doi: 10.1158/0008-5472.CAN-10-3894 21415164

[41] Rysman E Brusselmans K Scheys K Timmermans L Derua R Munck S. *De Novo* Lipogenesis Protects Cancer Cells From Free Radicals and Chemotherapeutics by Promoting Membrane Lipid Saturation. Cancer Res (2010) 70:8117–26. doi: 10.1158/0008-5472.Can-09-3871 20876798

[42] Kamphorst JJ Cross JR Fan J De Stanchina E Mathew R White EP. Hypoxic and Ras-Transformed Cells Support Growth by Scavenging Unsaturated Fatty Acids From Lysophospholipids. Proc Natl Acad Sci (2013) 110:8882–7. doi: 10.1073/pnas.1307237110 PMC3670379 23671091

[43] Beloribi-Djefaflia S Vasseur S Guillaumond F. Lipid Metabolic Reprogramming in Cancer Cells. Oncogenesis (2016) 5:e189. doi: 10.1038/oncsis.2015.49 26807644 PMC4728678

[44] Tabe Y Yamamoto S Saitoh K Sekihara K Monma N Ikeo K. Bone Marrow Adipocytes Facilitate Fatty Acid Oxidation Activating AMPK and a Transcriptional Network Supporting Survival of Acute Monocytic Leukemia Cells. Cancer Res (2017) 77:1453–64. doi: 10.1158/0008-5472.CAN-16-1645 PMC5354955 28108519

[45] Benesch MG Ko YM Tang X Dewald J Lopez-Campistrous A Zhao YY. Autotaxin is an Inflammatory Mediator and Therapeutic Target in Thyroid Cancer. Endocrine-Related Cancer (2015) 22:593–607. doi: 10.1530/erc-15-0045 26037280

[46] Magkrioti C Oikonomou N Kaffe E Mouratis M-A Xylourgidis N Barbayianni I. The Autotaxin—Lysophosphatidic Acid Axis Promotes Lung Carcinogenesis. Cancer Res (2018) 78:3634–44. doi: 10.1158/0008-5472.CAN-17-3797 29724718

[47] Shao Y Yu Y He Y Chen Q Liu H. Serum ATX as a Novel Biomarker for Breast Cancer. Medicine (2019) 98:e14973. doi: 10.1097/MD.0000000000014973 30921203 PMC6456097

[48] Memet I Tsalkidou E Tsaroucha AK Lambropoulou M Chatzaki E Trypsianis G. Autotaxin Expression in Hepatocellular Carcinoma. J Invest Surgery (2018) 31:359–65. doi: 10.1080/08941939.2017.1331280 28598712

[49] Nakai Y Ikeda H Nakamura K Kume Y Fujishiro M Sasahira N. Specific Increase in Serum Autotaxin Activity in Patients With Pancreatic Cancer. Clin Biochem (2011) 44:576–81. doi: 10.1016/j.clinbiochem.2011.03.128 21439952

[50] Xu A Ahsanul Kabir Khan M Chen F Zhong Z Chen HC Song Y. Overexpression of Autotaxin Is Associated With Human Renal Cell Carcinoma and Bladder Carcinoma and Their Progression. Med Oncol (Northwood London England) (2016) 33:131. doi: 10.1007/s12032-016-0836-7 27757783

[51] Nouh MAAM Wu XX Okazoe H Tsunemori H Haba R Abou-Zeid AMM. Expression of Autotaxin and Acylglycerol Kinase in Prostate Cancer: Association With Cancer Development and Progression. Cancer Sci (2009) 100:1631–8. doi: 10.1111/j.1349-7006.2009.01234.x PMC11158477 19549252

[52] Seo EJ Kwon YW Jang IH Kim DK Lee SI Choi EJ. Autotaxin Regulates Maintenance of Ovarian Cancer Stem Cells Through Lysophosphatidic Acid-Mediated Autocrine Mechanism. Stem Cells (Dayton Ohio) (2016) 34:551–64. doi: 10.1002/stem.2279 26800320

[53] Zhang G Cheng Y Zhang Q Li X Zhou J Wang J. ATX−LPA Axis Facilitates Estrogen−Induced Endometrial Cancer Cell Proliferation *via* MAPK/ERK Signaling Pathway. Mol Med Rep (2018) 17:4245–52. doi: 10.3892/mmr.2018.8392 PMC5802196 29328374

[54] Mazzocca A Schönauer LM De Nola R Lippolis A Marrano T Loverro M. Autotaxin Is a Novel Molecular Identifier of Type I Endometrial Cancer. Med Oncol (2018) 35:1–8. doi: 10.1007/s12032-018-1222-4 30374843

[55] Azare J Doane A Leslie K Chang Q Berishaj M Nnoli J. Stat3 Mediates Expression of Autotaxin in Breast Cancer. PloS One (2011) 6:e27851. doi: 10.1371/journal.pone.0027851 22140473 PMC3225372

[56] Yang L Yu X Yang Y. Autotaxin Upregulated by STAT3 Activation Contributes to Invasion in Pancreatic Neuroendocrine Neoplasms. Endocrine Connections (2018) 7:1299–307. doi: 10.1530/EC-18-0356 PMC6240148 30352421

[57] Zuckerman V Sokolov E Swet JH Ahrens WA Showlater V Iannitti DA. Expression and Function of Lysophosphatidic Acid Receptors (LPARs) 1 and 3 in Human Hepatic Cancer Progenitor Cells. Oncotarget (2016) 7:2951. doi: 10.18632/oncotarget.6696 26701886 PMC4823083

[58] Willier S Butt E Grunewald TG. Lysophosphatidic Acid (LPA) Signalling in Cell Migration and Cancer Invasion: A Focussed Review and Analysis of LPA Receptor Gene Expression on the Basis of More Than 1700 Cancer Microarrays. Biol Cell (2013) 105:317–33. doi: 10.1111/boc.201300011 23611148

[59] Kim EK Yun SJ Do KH Kim MS Cho M Suh D-S. Lysophosphatidic Acid Induces Cell Migration Through the Selective Activation of Akt1. Exp Mol Med (2008) 40:445–52. doi: 10.3858/emm.2008.40.4.445 PMC2679274 18779657

[60] Herr DR. Potential Use of G Protein-Coupled Receptor-Blocking Monoclonal Antibodies as Therapeutic Agents for Cancers. Int Rev Cell Mol Biol (2012) 297:45–81. doi: 10.1016/B978-0-12-394308-8.00002-9 22608557

[61] Yung YC Stoddard NC Chun J. LPA Receptor Signaling: Pharmacology, Physiology, and Pathophysiology. J Lipid Res (2014) 55:1192–214. doi: 10.1194/jlr.R046458 PMC4076099 24643338

[62] Samadi N Bekele R Capatos D Venkatraman G Sariahmetoglu M Brindley DN. Regulation of Lysophosphatidate Signaling by Autotaxin and Lipid Phosphate Phosphatases With Respect to Tumor Progression, Angiogenesis, Metastasis and Chemo-Resistance. Biochimie (2011) 93:61–70. doi: 10.1016/j.biochi.2010.08.002 20709140

[63] Tigyi GJ Yue J Norman DD Szabo E Balogh A Balazs L. Regulation of Tumor Cell–Microenvironment Interaction by the Autotaxin-Lysophosphatidic Acid Receptor Axis. Adv Biol Regulation (2019) 71:183–93. doi: 10.1016/j.jbior.2018.09.008 PMC6433480 30243984

[64] Shida D Kitayama J Yamaguchi H Hama K Aoki J Arai H. Dual Mode Regulation of Migration by Lysophosphatidic Acid in Human Gastric Cancer Cells. Exp Cell Res (2004) 301:168–78. doi: 10.1016/j.yexcr.2004.08.008 15530853

[65] Sahay D Leblanc R Grunewald TG Ambatipudi S Ribeiro J Clézardin P. The LPA1/ZEB1/miR-21-Activation Pathway Regulates Metastasis in Basal Breast Cancer. Oncotarget (2015) 6:20604. doi: 10.18632/oncotarget.3774 26098771 PMC4653029

[66] Shida D Kitayama J Yamaguchi H Okaji Y Tsuno NH Watanabe T. Lysophosphatidic Acid (LPA) Enhances the Metastatic Potential of Human Colon Carcinoma DLD1 Cells Through LPA1. Cancer Res (2003) 63:1706–11. 12670925

[67] Li TT Alemayehu M Aziziyeh AI Pape C Pampillo M Postovit L-M. β-Arrestin/Ral Signaling Regulates Lysophosphatidic Acid–Mediated Migration and Invasion of Human Breast Tumor Cells. Mol Cancer Res (2009) 7:1064–77. doi: 10.1158/1541-7786.MCR-08-0578 19609003

[68] Lee J Park SY Lee EK Park CG Chung HC Rha SY. Activation of Hypoxia-Inducible Factor-1α Is Necessary for Lysophosphatidic Acid–Induced Vascular Endothelial Growth Factor Expression. Clin Cancer Res (2006) 12:6351–8. doi: 10.1158/1078-0432.CCR-06-1252 17085645

[69] Hashimoto S Mikami S Sugino H Yoshikawa A Hashimoto A Onodera Y. Lysophosphatidic Acid Activates Arf6 to Promote the Mesenchymal Malignancy of Renal Cancer. Nat Commun (2016) 7:1–11. doi: 10.1038/ncomms10656 PMC4748122 26854204

[70] Zhang H Bialkowska A Rusovici R Chanchevalap S Shim H Katz JP. Lysophosphatidic Acid Facilitates Proliferation of Colon Cancer Cells *via* Induction of Krüppel-Like Factor 5. J Biol Chem (2007) 282:15541–9. doi: 10.1074/jbc.M700702200 PMC2000347 17430902

[71] Sun K Cai H Duan X Yang Y Li M Qu J. Aberrant Expression and Potential Therapeutic Target of Lysophosphatidic Acid Receptor 3 in Triple-Negative Breast Cancers. Clin Exp Med (2015) 15:371–80. doi: 10.1007/s10238-014-0306-5 PMC4522273 25209561

[72] Yang M Zhong WW Srivastava N Slavin A Yang J Hoey T. G Protein-Coupled Lysophosphatidic Acid Receptors Stimulate Proliferation of Colon Cancer Cells Through the β-Catenin Pathway. Proc Natl Acad Sci (2005) 102:6027–32. doi: 10.1073/pnas.0501535102 PMC1087935 15837931

[73] Kato K Yoshikawa K Tanabe E Kitayoshi M Fukui R Fukushima N. Opposite Roles of LPA 1 and LPA 3 on Cell Motile and Invasive Activities of Pancreatic Cancer Cells. Tumor Biol (2012) 33:1739–44. doi: 10.1007/s13277-012-0433-0 22678979

[74] Harper K Arsenault D Boulay-Jean S Lauzier A Lucien F Dubois CM. Autotaxin Promotes Cancer Invasion *via* the Lysophosphatidic Acid Receptor 4: Participation of the Cyclic AMP/EPAC/Rac1 Signaling Pathway in Invadopodia Formation. Cancer Res (2010) 70:4634–43. doi: 10.1158/0008-5472.CAN-09-3813 20484039

[75] Ishii S Hirane M Fukushima K Tomimatsu A Fukushima N Tsujiuchi T. Diverse Effects of LPA4, LPA5 and LPA6 on the Activation of Tumor Progression in Pancreatic Cancer Cells. Biochem Biophys Res Commun (2015) 461:59–64. doi: 10.1016/j.bbrc.2015.03.169 25849892

[76] Mazzocca A Dituri F De Santis F Filannino A Lopane C Betz RC. Lysophosphatidic Acid Receptor LPAR6 Supports the Tumorigenicity of Hepatocellular Carcinoma. Cancer Res (2015) 75:532–43. doi: 10.1158/0008-5472.CAN-14-1607 25589345

[77] Lee S-C Fujiwara Y Liu J Yue J Shimizu Y Norman DD. Autotaxin and LPA1 and LPA5 Receptors Exert Disparate Functions in Tumor Cells *Versus* the Host Tissue Microenvironment in Melanoma Invasion and Metastasis. Mol Cancer Res (2015) 13:174–85. doi: 10.1158/1541-7786.MCR-14-0263 PMC4297753 25158955

[78] Takahashi K Fukushima K Onishi Y Inui K Node Y Fukushima N. Lysophosphatidic Acid (LPA) Signaling *via* LPA4 and LPA6 Negatively Regulates Cell Motile Activities of Colon Cancer Cells. Biochem Biophys Res Commun (2017) 483:652–7. doi: 10.1016/j.bbrc.2016.12.088 27993681

[79] Matayoshi S Chiba S Lin Y Arakaki K Matsumoto H Nakanishi T. Lysophosphatidic Acid Receptor 4 Signaling Potentially Modulates Malignant Behavior in Human Head and Neck Squamous Cell Carcinoma Cells. Int J Oncol (2013) 42:1560–8. doi: 10.3892/ijo.2013.1849 PMC3661186 23467751

[80] Jongsma M Matas-Rico E Rzadkowski A Jalink K Moolenaar WH. LPA Is a Chemorepellent for B16 Melanoma Cells: Action Through the cAMP-Elevating LPA5 Receptor. PloS One (2011) 6:e29260. doi: 10.1371/journal.pone.0029260 22195035 PMC3237609

[81] Takahashi K Fukushima K Otagaki S Ishimoto K Minami K Fukushima N. Effects of LPA1 and LPA6 on the Regulation of Colony Formation Activity in Colon Cancer Cells Treated With Anticancer Drugs. J Receptors Signal Transduction (2018) 38:71–5. doi: 10.1080/10799893.2018.1426608 29369010

[82] Zhao Y Shao Q Peng G. Exhaustion and Senescence: Two Crucial Dysfunctional States of T Cells in the Tumor Microenvironment. Cell Mol Immunol (2020) 17:27–35. doi: 10.1038/s41423-019-0344-8 31853000 PMC6952436

[83] Pardoll DM. The Blockade of Immune Checkpoints in Cancer Immunotherapy. Nat Rev Cancer (2012) 12:252–64. doi: 10.1038/nrc3239 PMC4856023 22437870

[84] Mortezaee K. Immune Escape: A Critical Hallmark in Solid Tumors. Life Sci (2020) 258:118110. doi: 10.1016/j.lfs.2020.118110 32698074

[85] Resh MD. Palmitoylation of Proteins in Cancer. Biochem Soc Trans (2017) 45:409–16. doi: 10.1042/bst20160233 28408481

[86] Chen M Andreozzi M Pockaj B Barrett MT Ocal IT McCullough AE. Development and Validation of a Novel Clinical Fluorescence *In Situ* Hybridization Assay to Detect JAK2 and PD-L1 Amplification: A Fluorescence *In Situ* Hybridization Assay for JAK2 and PD-L1 Amplification. Modern Pathol an Off J United States Can Acad Pathol Inc (2017) 30:1516–26. doi: 10.1038/modpathol.2017.86 28752839

[87] Yao H Lan J Li C Shi H Brosseau JP Wang H. Inhibiting PD-L1 Palmitoylation Enhances T-Cell Immune Responses Against Tumours. Nat Biomed Eng (2019) 3:306–17. doi: 10.1038/s41551-019-0375-6 30952982

[88] Yang Y Hsu JM Sun L Chan LC Li CW Hsu JL. Palmitoylation Stabilizes PD-L1 to Promote Breast Tumor Growth. Cell Res (2019) 29:83–6. doi: 10.1038/s41422-018-0124-5 PMC6318320 30514902

[89] Chen DS Mellman I. Oncology Meets Immunology: The Cancer-Immunity Cycle. Immunity (2013) 39:1–10. doi: 10.1016/j.immuni.2013.07.012 23890059

[90] Motz GT Coukos G. Deciphering and Reversing Tumor Immune Suppression. Immunity (2013) 39:61–73. doi: 10.1016/j.immuni.2013.07.005 23890064 PMC3782392

[91] Maan M Peters JM Dutta M Patterson AD. Lipid Metabolism and Lipophagy in Cancer. Biochem Biophys Res Commun (2018) 504:582–9. doi: 10.1016/j.bbrc.2018.02.097 PMC6086774 29438712

[92] Zhang Y Kurupati R Liu L Zhou XY Zhang G Hudaihed A. Enhancing CD8(+) T Cell Fatty Acid Catabolism Within a Metabolically Challenging Tumor Microenvironment Increases the Efficacy of Melanoma Immunotherapy. Cancer Cell (2017) 32:377–391.e9. doi: 10.1016/j.ccell.2017.08.004 28898698 PMC5751418

[93] Patsoukis N Bardhan K Chatterjee P Sari D Liu B Bell LN. PD-1 Alters T-Cell Metabolic Reprogramming by Inhibiting Glycolysis and Promoting Lipolysis and Fatty Acid Oxidation. Nat Commun (2015) 6:6692. doi: 10.1038/ncomms7692 25809635 PMC4389235

[94] Zhang C Yue C Herrmann A Song J Egelston C Wang T. STAT3 Activation-Induced Fatty Acid Oxidation in CD8(+) T Effector Cells Is Critical for Obesity-Promoted Breast Tumor Growth. Cell Metab (2020) 31:148–161.e5. doi: 10.1016/j.cmet.2019.10.013 31761565 PMC6949402

[95] Brown RE Steele RW Marmer DJ Hudson JL Brewster MA. Fatty Acids and the Inhibition of Mitogen-Induced Lymphocyte Transformation by Leukemic Serum. J Immunol (Baltimore Md. 1950) (1983) 131:1011–6. 6575097

[96] Kleinfeld AM Okada C. Free Fatty Acid Release From Human Breast Cancer Tissue Inhibits Cytotoxic T-Lymphocyte-Mediated Killing. J Lipid Res (2005) 46:1983–90. doi: 10.1194/jlr.M500151-JLR200 15961785

[97] Chowdhury PS Chamoto K Kumar A Honjo T. PPAR-Induced Fatty Acid Oxidation in T Cells Increases the Number of Tumor-Reactive CD8(+) T Cells and Facilitates Anti-PD-1 Therapy. Cancer Immunol Res (2018) 6:1375–87. doi: 10.1158/2326-6066.Cir-18-0095 30143538

[98] Saibil SD Paul MS Laister RC Garcia-Batres CR Israni-Winger K Elford AR. Activation of Peroxisome Proliferator-Activated Receptors α and δ Synergizes With Inflammatory Signals to Enhance Adoptive Cell Therapy. Cancer Res (2019) 79:445–51. doi: 10.1158/0008-5472.CAN-17-3053 30573521

[99] Ma X Bi E Lu Y Su P Huang C Liu L. Cholesterol Induces CD8(+) T Cell Exhaustion in the Tumor Microenvironment. Cell Metab (2019) 30:143–156.e5. doi: 10.1016/j.cmet.2019.04.002 31031094 PMC7061417

[100] Yang W Bai Y Xiong Y Zhang J Chen S Zheng X. Potentiating the Antitumour Response of CD8(+) T Cells by Modulating Cholesterol Metabolism. Nature (2016) 531:651–5. doi: 10.1038/nature17412 PMC4851431 26982734

[101] Martinet L Garrido I Filleron T Le Guellec S Bellard E Fournie JJ. Human Solid Tumors Contain High Endothelial Venules: Association With T- and B-Lymphocyte Infiltration and Favorable Prognosis in Breast Cancer. Cancer Res (2011) 71:5678–87. doi: 10.1158/0008-5472.Can-11-0431 21846823

[102] Mathew D Kremer KN Strauch P Tigyi G Pelanda R Torres RM. LPA(5) Is an Inhibitory Receptor That Suppresses CD8 T-Cell Cytotoxic Function *via* Disruption of Early TCR Signaling. Front Immunol (2019) 10:1159. doi: 10.3389/fimmu.2019.01159 31231367 PMC6558414

[103] Oda SK Strauch P Fujiwara Y Al-Shami A Oravecz T Tigyi G. Lysophosphatidic Acid Inhibits CD8 T Cell Activation and Control of Tumor Progression. Cancer Immunol Res (2013) 1:245–55. doi: 10.1158/2326-6066.Cir-13-0043-t PMC3893823 24455753

[104] Basingab FS Ahmadi M Morgan DJ. Ifnγ-Dependent Interactions Between ICAM-1 and LFA-1 Counteract Prostaglandin E2-Mediated Inhibition of Antitumor CTL Responses. Cancer Immunol Res (2016) 4:400–11. doi: 10.1158/2326-6066.Cir-15-0146 26928462

[105] Howie D Cobbold SP Adams E Ten Bokum A Necula AS Zhang W. Foxp3 Drives Oxidative Phosphorylation and Protection From Lipotoxicity. JCI Insight (2017) 2:e89160. doi: 10.1172/jci.insight.89160 28194435 PMC5291728

[106] Michalek RD Gerriets VA Jacobs SR Macintyre AN MacIver NJ Mason EF. Cutting Edge: Distinct Glycolytic and Lipid Oxidative Metabolic Programs Are Essential for Effector and Regulatory CD4+ T Cell Subsets. J Immunol (Baltimore Md. 1950) (2011) 186:3299–303. doi: 10.4049/jimmunol.1003613 PMC3198034 21317389

[107] Pacella I Procaccini C Focaccetti C Miacci S Timperi E Faicchia D. Fatty Acid Metabolism Complements Glycolysis in the Selective Regulatory T Cell Expansion During Tumor Growth. Proc Natl Acad Sci USA (2018) 115:E6546–e6555. doi: 10.1073/pnas.1720113115 29941600 PMC6048537

[108] Angelin A Gil-de-Gómez L Dahiya S Jiao J Guo L Levine MH. Foxp3 Reprograms T Cell Metabolism to Function in Low-Glucose, High-Lactate Environments. Cell Metab (2017) 25:1282–1293.e7. doi: 10.1016/j.cmet.2016.12.018 28416194 PMC5462872

[109] Wang H Franco F Tsui Y-C Xie X Trefny MP Zappasodi R. CD36-Mediated Metabolic Adaptation Supports Regulatory T Cell Survival and Function in Tumors. Nat Immunol (2020) 21:298–308. doi: 10.1038/s41590-019-0589-5 32066953 PMC7043937

[110] Cassetta L Fragkogianni S Sims AH Swierczak A Forrester LM Zhang H. Human Tumor-Associated Macrophage and Monocyte Transcriptional Landscapes Reveal Cancer-Specific Reprogramming, Biomarkers, and Therapeutic Targets. Cancer Cell (2019) 35:588–602.e10. doi: 10.1016/j.ccell.2019.02.009 30930117 PMC6472943

[111] Mantovani A Sozzani S Locati M Allavena P Sica A. Macrophage Polarization: Tumor-Associated Macrophages as a Paradigm for Polarized M2 Mononuclear Phagocytes. Trends Immunol (2002) 23:549–55. doi: 10.1016/s1471-4906(02)02302-5 12401408

[112] Wang F Zhang S Vuckovic I Jeon R Lerman A Folmes CD. Glycolytic Stimulation Is Not a Requirement for M2 Macrophage Differentiation. Cell Metab (2018) 28:463–475.e4. doi: 10.1016/j.cmet.2018.08.012 30184486 PMC6449248

[113] Cook J Hagemann T. Tumour-Associated Macrophages and Cancer. Curr Opin Pharmacol (2013) 13:595–601. doi: 10.1016/j.coph.2013.05.017 23773801

[114] Su P Wang Q Bi E Ma X Liu L Yang M. Enhanced Lipid Accumulation and Metabolism Are Required for the Differentiation and Activation of Tumor-Associated Macrophages. Cancer Res (2020) 80:1438–50. doi: 10.1158/0008-5472.Can-19-2994 PMC7127942 32015091

[115] Namgaladze D Brüne B. Fatty Acid Oxidation Is Dispensable for Human Macrophage IL-4-Induced Polarization. Biochim Biophys Acta (2014) 1841:1329–35. doi: 10.1016/j.bbalip.2014.06.007 24960101

[116] Goossens P Rodriguez-Vita J Etzerodt A Masse M Rastoin O Gouirand V. Membrane Cholesterol Efflux Drives Tumor-Associated Macrophage Reprogramming and Tumor Progression. Cell Metab (2019) 29:1376–1389.e4. doi: 10.1016/j.cmet.2019.02.016 30930171

[117] Wang Z Brandt S Medeiros A Wang S Wu H Dent A. MicroRNA 21 Is a Homeostatic Regulator of Macrophage Polarization and Prevents Prostaglandin E2-Mediated M2 Generation. PloS One (2015) 10:e0115855. doi: 10.1371/journal.pone.0115855 25706647 PMC4338261

[118] Oishi Y Spann NJ Link VM Muse ED Strid T Edillor C. SREBP1 Contributes to Resolution of Pro-Inflammatory TLR4 Signaling by Reprogramming Fatty Acid Metabolism. Cell Metab (2017) 25:412–27. doi: 10.1016/j.cmet.2016.11.009 PMC5568699 28041958

[119] Schumann T Adhikary T Wortmann A Finkernagel F Lieber S Schnitzer E. Deregulation of Pparβ/δ Target Genes in Tumor-Associated Macrophages by Fatty Acid Ligands in the Ovarian Cancer Microenvironment. Oncotarget (2015) 6:13416–33. doi: 10.18632/oncotarget.3826 PMC4537024 25968567

[120] Podgornik H Sok M Kern I Marc J Cerne D. Lipoprotein Lipase in non-Small Cell Lung Cancer Tissue Is Highly Expressed in a Subpopulation of Tumor-Associated Macrophages. Pathol Res Practice (2013) 209:516–20. doi: 10.1016/j.prp.2013.06.004 23880163

[121] Xiu F Diao L Qi P Catapano M Jeschke MG. Palmitate Differentially Regulates the Polarization of Differentiating and Differentiated Macrophages. Immunology (2016) 147:82–96. doi: 10.1111/imm.12543 26453839 PMC4693883

[122] Thyagarajan N Marshall JD Pickett AT Schumacher C Yang Y Christian SL. Transcriptomic Analysis of THP-1 Macrophages Exposed to Lipoprotein Hydrolysis Products Generated by Lipoprotein Lipase. Lipids (2017) 52:189–205. doi: 10.1007/s11745-017-4238-1 28205069

[123] Zhang Q Wang H Mao C Sun M Dominah G Chen L. Fatty Acid Oxidation Contributes to IL-1β Secretion in M2 Macrophages and Promotes Macrophage-Mediated Tumor Cell Migration. Mol Immunol (2018) 94:27–35. doi: 10.1016/j.molimm.2017.12.011 29248877 PMC5801116

[124] Zhang Y Sun Y Rao E Yan F Li Q Zhang Y. Fatty Acid-Binding Protein E-FABP Restricts Tumor Growth by Promoting IFN-β Responses in Tumor-Associated Macrophages. Cancer Res (2014) 74:2986–98. doi: 10.1158/0008-5472.Can-13-2689 PMC4040301 24713431

[125] Chiba S Hisamatsu T Suzuki H Mori K Kitazume MT Shimamura K. Glycolysis Regulates LPS-Induced Cytokine Production in M2 Polarized Human Macrophages. Immunol Lett (2017) 183:17–23. doi: 10.1016/j.imlet.2017.01.012 28130076

[126] Rice CM Davies LC Subleski JJ Maio N Gonzalez-Cotto M Andrews C. Tumour-Elicited Neutrophils Engage Mitochondrial Metabolism to Circumvent Nutrient Limitations and Maintain Immune Suppression. Nat Commun (2018) 9:5099. doi: 10.1038/s41467-018-07505-2 30504842 PMC6269473

[127] Brandau S Moses K Lang S. The Kinship of Neutrophils and Granulocytic Myeloid-Derived Suppressor Cells in Cancer: Cousins, Siblings or Twins? Semin Cancer Biol (2013) 23:171–82. doi: 10.1016/j.semcancer.2013.02.007 23459190

[128] Ost M Singh A Peschel A Mehling R Rieber N Hartl D. Myeloid-Derived Suppressor Cells in Bacterial Infections. Front Cell Infect Microbiol (2016) 6:37. doi: 10.3389/fcimb.2016.00037 27066459 PMC4814452

[129] Ye Y Sun X Lu Y. Obesity-Related Fatty Acid and Cholesterol Metabolism in Cancer-Associated Host Cells. Front Cell Dev Biol (2020) 8:1149. doi: 10.3389/fcell.2020.600350 PMC7729017 33330490

[130] Hossain F Al-Khami AA Wyczechowska D Hernandez C Zheng L Reiss K. Inhibition of Fatty Acid Oxidation Modulates Immunosuppressive Functions of Myeloid-Derived Suppressor Cells and Enhances Cancer Therapies. Cancer Immunol Res (2015) 3:1236–47. doi: 10.1158/2326-6066.Cir-15-0036 PMC4636942 26025381

[131] Rong Y Yuan C-H Qu Z Zhou H Guan Q Yang N. Doxorubicin Resistant Cancer Cells Activate Myeloid-Derived Suppressor Cells by Releasing PGE 2. Sci Rep (2016) 6:1–11. doi: 10.1038/srep23824 27032536 PMC4817121

[132] Pearce EJ Everts B. Dendritic Cell Metabolism. Nat Rev Immunol (2015) 15:18–29. doi: 10.1038/nri3771 25534620 PMC4495583

[133] Herber DL Cao W Nefedova Y Novitskiy SV Nagaraj S Tyurin VA. Lipid Accumulation and Dendritic Cell Dysfunction in Cancer. Nat Med (2010) 16:880–6. doi: 10.1038/nm.2172 PMC2917488 20622859

[134] Cubillos-Ruiz JR Silberman PC Rutkowski MR Chopra S Perales-Puchalt A Song M. ER Stress Sensor XBP1 Controls Anti-Tumor Immunity by Disrupting Dendritic Cell Homeostasis. Cell (2015) 161:1527–38. doi: 10.1016/j.cell.2015.05.025 PMC4580135 26073941

[135] Veglia F Tyurin VA Mohammadyani D Blasi M Duperret EK Donthireddy L. Lipid Bodies Containing Oxidatively Truncated Lipids Block Antigen Cross-Presentation by Dendritic Cells in Cancer. Nat Commun (2017) 8:2122. doi: 10.1038/s41467-017-02186-9 29242535 PMC5730553

[136] Ramakrishnan R Tyurin VA Veglia F Condamine T Amoscato A Mohammadyani D. Oxidized Lipids Block Antigen Cross-Presentation by Dendritic Cells in Cancer. J Immunol (Baltimore Md. 1950) (2014) 192:2920–31. doi: 10.4049/jimmunol.1302801 PMC3998104 24554775

[137] Gao F Liu C Guo J Sun W Xian L Bai D. Radiation-Driven Lipid Accumulation and Dendritic Cell Dysfunction in Cancer. Sci Rep (2015) 5:9613. doi: 10.1038/srep09613 25923834 PMC4413852

[138] Malinarich F Duan K Hamid RA Bijin A Lin WX Poidinger M. High Mitochondrial Respiration and Glycolytic Capacity Represent a Metabolic Phenotype of Human Tolerogenic Dendritic Cells. J Immunol (Baltimore Md. 1950) (2015) 194:5174–86. doi: 10.4049/jimmunol.1303316 25917094

[139] Plebanek MP Sturdivant M DeVito NC Hanks BA. Role of Dendritic Cell Metabolic Reprogramming in Tumor Immune Evasion. Int Immunol (2020) 32:485–91. doi: 10.1093/intimm/dxaa036 PMC7318778 32449776

[140] Viel S Besson L Charrier E Marçais A Disse E Bienvenu J. Alteration of Natural Killer Cell Phenotype and Function in Obese Individuals. Clin Immunol (Orlando Fla.) (2017) 177:12–7. doi: 10.1016/j.clim.2016.01.007 26794911

[141] Yaqoob P Newsholme EA Calder PC. Inhibition of Natural Killer Cell Activity by Dietary Lipids. Immunol Lett (1994) 41:241–7. doi: 10.1016/0165-2478(94)90140-6 8002045

[142] Tobin LM Mavinkurve M Carolan E Kinlen D O'Brien EC Little MA. NK Cells in Childhood Obesity Are Activated, Metabolically Stressed, and Functionally Deficient. JCI Insight (2017) 2:e94939. doi: 10.1172/jci.insight.94939 PMC5752310 29263296

[143] Donnelly RP Loftus RM Keating SE Liou KT Biron CA Gardiner CM. Mtorc1-Dependent Metabolic Reprogramming Is a Prerequisite for NK Cell Effector Function. J Immunol (Baltimore Md. 1950) (2014) 193:4477–84. doi: 10.4049/jimmunol.1401558 PMC4201970 25261477

[144] Park A Lee Y Kim MS Kang YJ Park Y-J Jung H. Prostaglandin E2 Secreted by Thyroid Cancer Cells Contributes to Immune Escape Through the Suppression of Natural Killer (NK) Cell Cytotoxicity and NK Cell Differentiation. Front Immunol (2018) 9:1859. doi: 10.3389/fimmu.2018.01859 30140269 PMC6094168

[145] Ma X Holt D Kundu N Reader J Goloubeva O Take Y. A Prostaglandin E (PGE) Receptor EP4 Antagonist Protects Natural Killer Cells From PGE2-Mediated Immunosuppression and Inhibits Breast Cancer Metastasis. Oncoimmunology (2013) 2:e22647. doi: 10.4161/onci.22647 23482441 PMC3583931

[146] Böttcher JP Bonavita E Chakravarty P Blees H Cabeza-Cabrerizo M Sammicheli S. NK Cells Stimulate Recruitment of Cdc1 Into the Tumor Microenvironment Promoting Cancer Immune Control. Cell (2018) 172:1022–1037.e14. doi: 10.1016/j.cell.2018.01.004 29429633 PMC5847168

[147] Neelapu SS Locke FL Bartlett NL Lekakis LJ Miklos DB Jacobson CA. Axicabtagene Ciloleucel CAR T-Cell Therapy in Refractory Large B-Cell Lymphoma. N Engl J Med (2017) 377:2531–44. doi: 10.1056/NEJMoa1707447 PMC5882485 29226797

[148] Maude SL Frey N Shaw PA Aplenc R Barrett DM Bunin NJ. Chimeric Antigen Receptor T Cells for Sustained Remissions in Leukemia. N Engl J Med (2014) 371:1507–17. doi: 10.1056/NEJMoa1407222 PMC4267531 25317870

[149] Martinez M Moon EK. CAR T Cells for Solid Tumors: New Strategies for Finding, Infiltrating, and Surviving in the Tumor Microenvironment. Front Immunol (2019) 10:128. doi: 10.3389/fimmu.2019.00128 30804938 PMC6370640

[150] Dobrenkov K Ostrovnaya I Gu J Cheung IY Cheung NK. Oncotargets GD2 and GD3 Are Highly Expressed in Sarcomas of Children, Adolescents, and Young Adults. Pediatr Blood Cancer (2016) 63:1780–5. doi: 10.1002/pbc.26097 PMC5215083 27304202

[151] Louis CU Savoldo B Dotti G Pule M Yvon E Myers GD. Antitumor Activity and Long-Term Fate of Chimeric Antigen Receptor-Positive T Cells in Patients With Neuroblastoma. Blood (2011) 118:6050–6. doi: 10.1182/blood-2011-05-354449 PMC3234664 21984804

[152] Straathof K Flutter B Wallace R Jain N Loka T Depani S. Antitumor Activity Without on-Target Off-Tumor Toxicity of GD2-Chimeric Antigen Receptor T Cells in Patients With Neuroblastoma. Sci Trans Med (2020) 12:eabd6169. doi: 10.1126/scitranslmed.abd6169 33239386

[153] Heczey A Louis CU Savoldo B Dakhova O Durett A Grilley B. CAR T Cells Administered in Combination With Lymphodepletion and PD-1 Inhibition to Patients With Neuroblastoma. Mol Ther J Am Soc Gene Ther (2017) 25:2214–24. doi: 10.1016/j.ymthe.2017.05.012 PMC5589058 28602436

[154] Kondo T Ando M Nagai N Tomisato W Srirat T Liu B. The NOTCH-FOXM1 Axis Plays a Key Role in Mitochondrial Biogenesis in the Induction of Human Stem Cell Memory-Like CAR-T Cells. Cancer Res (2020) 80:471–83. doi: 10.1158/0008-5472.Can-19-1196 31767627

[155] Jiang L Fang X Wang H Li D Wang X. Ovarian Cancer-Intrinsic Fatty Acid Synthase Prevents Anti-Tumor Immunity by Disrupting Tumor-Infiltrating Dendritic Cells. Front Immunol (2018) 9:2927. doi: 10.3389/fimmu.2018.02927 30619288 PMC6302125

[156] Voss K Luthers CR Pohida K Snow AL. Fatty Acid Synthase Contributes to Restimulation-Induced Cell Death of Human CD4 T Cells. Front Mol Biosciences (2019) 6:106. doi: 10.3389/fmolb.2019.00106 PMC6803432 31681794

[157] Chamoto K Chowdhury PS Kumar A Sonomura K Matsuda F Fagarasan S. Mitochondrial Activation Chemicals Synergize With Surface Receptor PD-1 Blockade for T Cell-Dependent Antitumor Activity. Proc Natl Acad Sci USA (2017) 114:E761–70. doi: 10.1073/pnas.1620433114 28096382 PMC5293087

[158] Liu X Bao X Hu M Chang H Jiao M Cheng J. Inhibition of PCSK9 Potentiates Immune Checkpoint Therapy for Cancer. Nature (2020) 588:693–8. doi: 10.1038/s41586-020-2911-7 PMC7770056 33177715

[159] Omori M Okuma Y Hakozaki T Hosomi Y. Statins Improve Survival in Patients Previously Treated With Nivolumab for Advanced Non−Small Cell Lung Cancer: An Observational Study. Mol Clin Oncol (2019) 10:137–43. doi: 10.3892/mco.2018.1765 PMC6313973 30655989

[160] Cortellini A Tucci M Adamo V Stucci LS Russo A Tanda ET. Integrated Analysis of Concomitant Medications and Oncological Outcomes From PD-1/PD-L1 Checkpoint Inhibitors in Clinical Practice. J Immunother Cancer (2020) 8:e001361. doi: 10.1136/jitc-2020-001361 33154150 PMC7646355

[161] Cantini L Pecci F Hurkmans DP Belderbos RA Lanese A Copparoni C. High-Intensity Statins are Associated With Improved Clinical Activity of PD-1 Inhibitors in Malignant Pleural Mesothelioma and Advanced Non-Small Cell Lung Cancer Patients. Eur J Cancer (2021) 144:41–8. doi: 10.1016/j.ejca.2020.10.031 33326868

[162] Mock A Zschäbitz S Kirsten R Scheffler M Wolf B Herold-Mende C. Serum Very Long-Chain Fatty Acid-Containing Lipids Predict Response to Immune Checkpoint Inhibitors in Urological Cancers. Cancer Immunol Immunother CII (2019) 68:2005–14. doi: 10.1007/s00262-019-02428-3 PMC11028211 31701161

[163] Wilson BE Routy B Nagrial A Chin VT. The Effect of Antibiotics on Clinical Outcomes in Immune-Checkpoint Blockade: A Systematic Review and Meta-Analysis of Observational Studies. Cancer Immunol Immunother CII (2020) 69:343–54. doi: 10.1007/s00262-019-02453-2 PMC11027824 31865400

[164] Yu Y Zheng P Gao L Li H Tao P Wang D. Effects of Antibiotic Use on Outcomes in Cancer Patients Treated Using Immune Checkpoint Inhibitors: A Systematic Review and Meta-Analysis. J Immunother (Hagerstown Md. 1997) (2021) 44:76–85. doi: 10.1097/cji.0000000000000346 33208635

[165] Routy B Le Chatelier E Derosa L Duong CPM Alou MT Daillère R. Gut Microbiome Influences Efficacy of PD-1-Based Immunotherapy Against Epithelial Tumors. Sci (New York N.Y.) (2018) 359:91–7. doi: 10.1126/science.aan3706 29097494

[166] Lurienne L Cervesi J Duhalde L de Gunzburg J Andremont A Zalcman G. NSCLC Immunotherapy Efficacy and Antibiotic Use: A Systematic Review and Meta-Analysis. J Thorac Oncol Off Publ Int Assoc Study Lung Cancer (2020) 15:1147–59. doi: 10.1016/j.jtho.2020.03.002 32173463

[167] Nomura M Nagatomo R Doi K Shimizu J Baba K Saito T. Association of Short-Chain Fatty Acids in the Gut Microbiome With Clinical Response to Treatment With Nivolumab or Pembrolizumab in Patients With Solid Cancer Tumors. JAMA Network Open (2020) 3:e202895. doi: 10.1001/jamanetworkopen.2020.2895 32297948 PMC7163404

[168] Botticelli A Vernocchi P Marini F Quagliariello A Cerbelli B Reddel S. Gut Metabolomics Profiling of Non-Small Cell Lung Cancer (NSCLC) Patients Under Immunotherapy Treatment. J Trans Med (2020) 18:49. doi: 10.1186/s12967-020-02231-0 PMC6998840 32014010

[169] An Y Wu Z Wang N Yang Z Li Y Xu B. Association Between Body Mass Index and Survival Outcomes for Cancer Patients Treated With Immune Checkpoint Inhibitors: A Systematic Review and Meta-Analysis. J Trans Med (2020) 18:235. doi: 10.1186/s12967-020-02404-x PMC7291531 32532255

[170] Xu H Cao D He A Ge W. The Prognostic Role of Obesity is Independent of Sex in Cancer Patients Treated With Immune Checkpoint Inhibitors: A Pooled Analysis of 4090 Cancer Patients. Int Immunopharmacology (2019) 74:105745. doi: 10.1016/j.intimp.2019.105745 31302449

[171] Cuello MA Kato S Liberona F. The Impact on High-Grade Serous Ovarian Cancer of Obesity and Lipid Metabolism-Related Gene Expression Patterns: The Underestimated Driving Force Affecting Prognosis. J Cell Mol Med (2018) 22:1805–15. doi: 10.1111/jcmm.13463 PMC5824367 29266765

[172] Wu F Zhao Z Chai RC Liu YQ Li GZ Jiang HY. Prognostic Power of a Lipid Metabolism Gene Panel for Diffuse Gliomas. J Cell Mol Med (2019) 23:7741–8. doi: 10.1111/jcmm.14647 PMC6815778 31475440

