# Supplementary material for: A Forgotten Corner in Cancer Immunotherapy: The Role of Lipids
Source: Front Oncol. 2021 Oct 14;11:751086. doi: 10.3389/fonc.2021.751086 (PMC8551635; doi:10.3389/fonc.2021.751086)
Supplement: Supplementary file 1 [file DataSheet_1.docx]

**Search strategy for literatures in this review**

1. **Electronic search in PubMed**

#1 "Immunity"[Mesh]

#2 "Immune Checkpoint Inhibitors"[Mesh]

#3 “Immunotherapy, Active”[Mesh]

#4 “CTLA-4 Antigen”[Mesh]

#5 “Programmed Cell Death 1 Receptor”[Mesh]

#6 immunity[title/abstract]

#7 immune[title/abstract]

#8 immunotherapy[title/abstract]

#9 “checkpoint inhibitor”[title/abstract]

#10 CTLA-4[title/abstract]

#11 PD-1[title/abstract]

#12 PD-L1[title/abstract]

#13 #1-12 OR

#14 "Neoplasms"[Mesh]

#15 "Carcinoma"[Mesh]

#16 cancer[title/abstract]

#17 carcinoma[title/abstract]

#18 neoplasm[title/abstract]

#19 tumor[title/abstract]

#20 #14-19 OR

#21 "Lipids"[Mesh]

#22 "Lipid Metabolism"[Mesh]

#23 "Lipoproteins"[Mesh]

#24 "Cholesterol"[Mesh]

#25 lipid[tiab]

#26 lipoprotein[tiab]

#27 cholesterol[title/abstract]

#28 HDL[title/abstract]

#29 LDL[title/abstract]

#30 #21-29 OR

#31 #13 AND #20 AND #30

Limited to recent 5 years and English literatures.

The search results were sorted by “Best match” and the first 300 records were screened for eligible literatures.

1. **Additional search**

Google scholar was used to identify additional literatures that are not recorded in PubMed database. The reference lists of eligible literatures were also tracked and searched to obtain any potentially relevant publications.
